# Inhibition of human histone lysine methyltransferases by a redox-labile S-adenosyl-L-homocysteine analog

**DOI:** 10.1038/s41598-026-52984-9

**Published:** 2026-05-13

**Authors:** Faidra Voukia, Laust Moesgaard, Jacob Kongsted, Jasmin Mecinović

**Affiliations:** Department of Physics, Chemistry and Pharmacy, University of Southern Denmark, Campusvej 55, 5230 Odense, Denmark

**Keywords:** Biochemistry, Chemical biology, Computational biology and bioinformatics, Drug discovery

## Abstract

Methylation of lysine residues in histones by histone lysine methyltransferases is important for epigenetic gene regulation in humans. Histone lysine methyltransferases use S-adenosyl-L-methionine (SAM) as a co-substrate, producing the methylated lysine product and S-adenosyl-L-homocysteine (SAH) by-product. Inhibition of this class of epigenetic enzymes has potential for treatment of human diseases, including cancer. Here, we designed and developed a redox-labile S-adenosyl-L-homocysteine analog (**SS-SAH**) possessing a disulfide bond as a reversible inhibitor of biomedically important histone lysine methyltransferases GLP, G9a and SETD8. Combined enzyme assays, docking studies and molecular dynamics simulations provide insight into inhibition and binding mode of the redox-labile **SS-SAH**, and demonstrate that the inhibitory activity of **SS-SAH** could be deactivated by cleavage of the disulfide bond upon the addition of a reductive agent. Overall, this work highlights a utility of unique redox-labile inhibitors of epigenetic enzymes.

**Supplementary Information:**

The online version contains supplementary material available at 10.1038/s41598-026-52984-9.

## Introduction

S-adenosyl-L-methionine (SAM) is a co-factor ubiquitous in nature, where it participates as a methyl donor in the methylation of nucleophilic groups^1,2^. SAM-dependent methylation proceeds via an S_N_2 mechanism, where the nucleophilic atom attacks the electrophilic methyl group of SAM, under the catalysis of SAM-dependent methyltransferases^1,3^. SAM-dependent methyltransferases represent a diverse family of enzymes that catalyze the methylation of a vast array of substrates ranging from proteins, DNA and RNA, to small molecule metabolites^1–4^. Due to the diversity of substrates, methyltransferases have been a subject of biomedical research in the past few decades^3,4^.

**Fig. 1:**
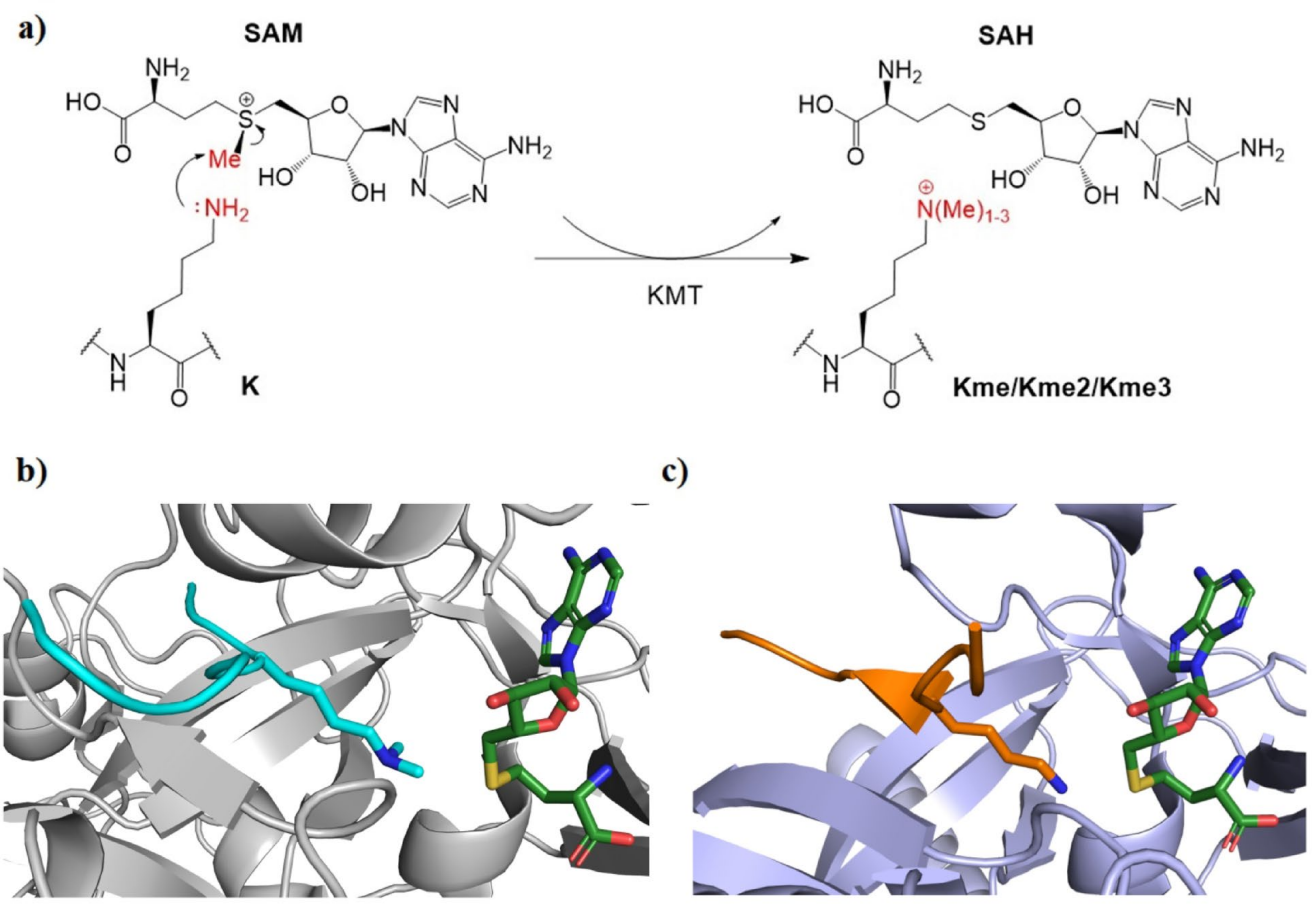
Lysine methylation by histone lysine methyltransferases. (**a**) KMT-catalyzed methylation of lysine residues in the presence of S-adenosyl-L-methionine (SAM), leading to methylated lysine residues and S-adenosyl-L-homocysteine (SAH) as the by-product. (**b**) View on the crystal structure of GLP (grey), complexed with SAH (green) and H3K9me2 (cyan) (PDB: 2RFI). (**c**) View on the crystal structure of SETD8 (blue), complexed with SAH (green) and H4K20 (orange) (PDB: 3F9W). Images in panels (**b**) and (**c**) were made in PyMOL Molecular Graphics System, Version 3.1 Schrödinger, LLC (https://www.pymol.org/).

Histone lysine methyltransferases (KMTs) are biomedically important epigenetic enzymes that catalyze methylation of lysine residues in histones, leading to a formation of mono-, di- and trimethyllysine (Kme, Kme2 and Kme3, respectively) (Fig. 1)^5–9^. Methylation of lysine residues in histones plays an important biological role in epigenetic gene regulation, and aberrant function of histone lysine methyltransferases is associated with human diseases, including cancer^10–14^. Due to the exceptional biomedical potential of methyltransferases, it is apparent that studying the structure and function of this superfamily of enzymes advances the biomedicinal opportunities in discovering selective and potent inhibitors^14–18^.

S-Adenosyl-L-homocysteine (SAH), the by-product of enzymatic SAM-dependent methylation, has been reported to have a high binding affinity for all SAM-dependent methyltransferases, while also acting as a potent SAM-competitive inhibitor for many of them (Fig. 1)^3,19,20^. For example, it has been found in vitro that SAH is a pan-inhibitor potent against, among others, DNA methyltransferase DNMT1 (IC_50_ = 0.34 µM), arginine methyltransferases (PRMTs, IC_50_ = 0.1–4.5 µM) and the RNA methyltransferase complex METTL3/METTL14 (IC_50_ = 1.11 µM)^21–23^. As a result, a well-established approach for inhibition of several methyltransferases has relied on synthetic SAH analogs^3,24–27^. Therefore, compounds that involve modifications on the SAH structure are of interest with the purpose of improving selectivity and potency^3,4,6^.

Varied chemical groups can be introduced into biologically active molecules, responding to different environmental changes, such as light, change in pH, or change in the redox potential^28^. The introduction of these chemical groups can be useful in inhibitors, probes and other ligands, making them valuable tools in many research applications in biochemical and biomedical fields^28,29^. The disulfide bond is an example of a chemical group sensitive to changes in the redox potential, as it can be smoothly cleaved in a reducing environment. This has been mainly applied as a mechanism for activatable chemical probes^30,31^ or prodrugs that become active drug compounds by disulfide bond cleavage^28,29^. The potential of using disulfides as deactivatable inhibitors, however, has been explored less frequently^32^.

**Fig. 2:**
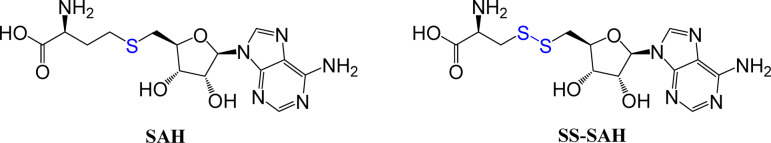
Structures of SAH and its redox-labile **SS-SAH** analog.

In this study, we designed and synthesized a redox-labile SAH analog containing a disulfide bond (**SS-SAH**, Fig. 2), and examined its inhibitory activity against selected human KMTs. We hypothesized that this potential KMT inhibitor could be deactivated on demand, by cleavage of a disulfide bond when a reductive agent is used. We envisioned that exploiting this flexibility and immediate control over the enzymatic activity could have useful applications in in vitro studies of biomedically important methyltransferases.

## Results and discussion

**Scheme 1:**
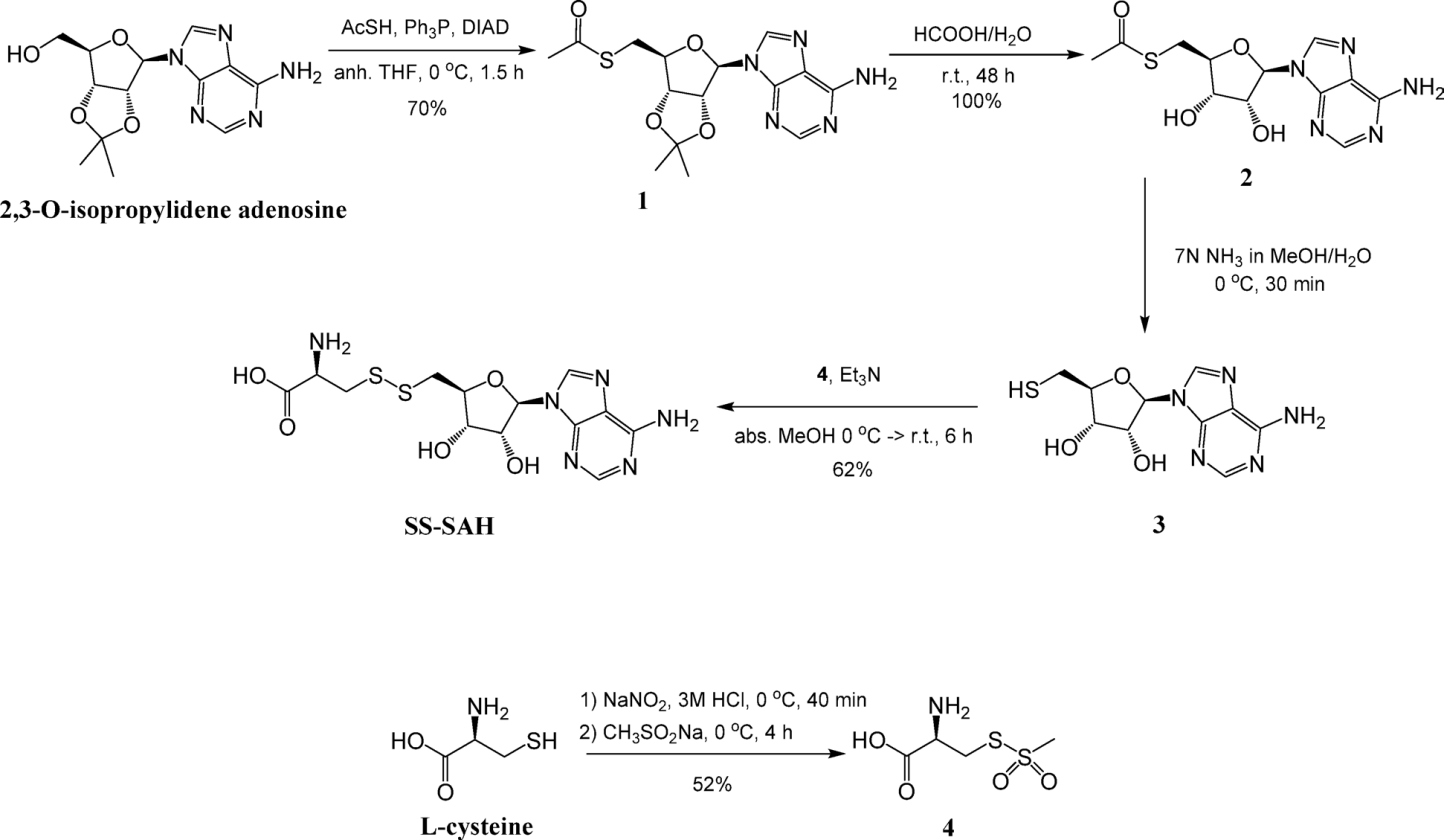
Synthesis of **SS-SAH**.

Because the SAH structure contains a thioether bond, we conceived that replacing a methylene group adjacent to the S atom in SAH would lead to the redox-labile functional mimic of SAH, hereafter named as **SS-SAH** (Fig. 2). The connection of atoms and the position of the disulfide bridge in **SS-SAH** result in a molecule that is stable and easily accessible via a multistep synthetic route from readily available starting materials. The redox-labile **SS-SAH** was synthesized using L-cysteine and 2′,3′-O-isopropylidene adenosine as starting materials (Scheme 1). Synthesis of thioester **1** was carried out from 2′,3′-O-isopropylidene adenosine and thioacetic acid under the Mitsunobu conditions (70% yield)^25^. The cyclic acetal of **1** was then deprotected with formic acid/water (1:1 v/v) to produce **2** in quantitative yield. Finally, the thioester was hydrolyzed by 7 N ammonia in MeOH/water, resulting in thiol **3**, which was used in the next step immediately without purification. Additionally, the thiol group of L-cysteine was activated with a one-pot nitrosylation and subsequent mesylation^33^, to produce **4** in 52% yield. Finally, **3** and **4** were reacted under basic conditions to afford **SS-SAH** in 62% yield. Compounds **1**–**4** and **SS-SAH** were characterized by ^1^H and ^13^C NMR (Figs. S1–S5), while **SS-SAH** was further analyzed by HPLC and ESI-HRMS (Figs. S6-S7).

**SS-SAH** is stable in buffers at physiological pH. We observed the degradation of **SS-SAH** under reduced conditions when **SS-SAH** (100 µM) was incubated with either 1 mM, 500 µM or 200 µM of reducing agent dithiothreitol (DTT) or 1 mM of glutathione (GSH) (a natural anti-oxidant with a concentration range of 1–10 mM in human cells)^29^ for 60 min in 50 mM Tris-HCl buffer, pH = 8.0. The degradation of **SS-SAH** was monitored by analytical HPLC at 254 nm, and it was immediately observed after adding the reductive agents (Figs. S8-S15). In the presence of DTT, the reduction of **SS-SAH** led to the formation of thiol **3**, as verified by HPLC analyses (Figs. S8-S10). LC-MS data further supported the DTT-mediated reduction of **SS-SAH** to thiol **3** (Fig. S11). Interestingly, in the presence of GSH, an additional peak was observed, corresponding to the asymmetric disulfide between thiol **3** and GSH (Figs. S12-S13). The formation of the **3**-GSH adduct was further verified by LC-MS analyses (Figs. S14-S15).

**Fig. 3:**
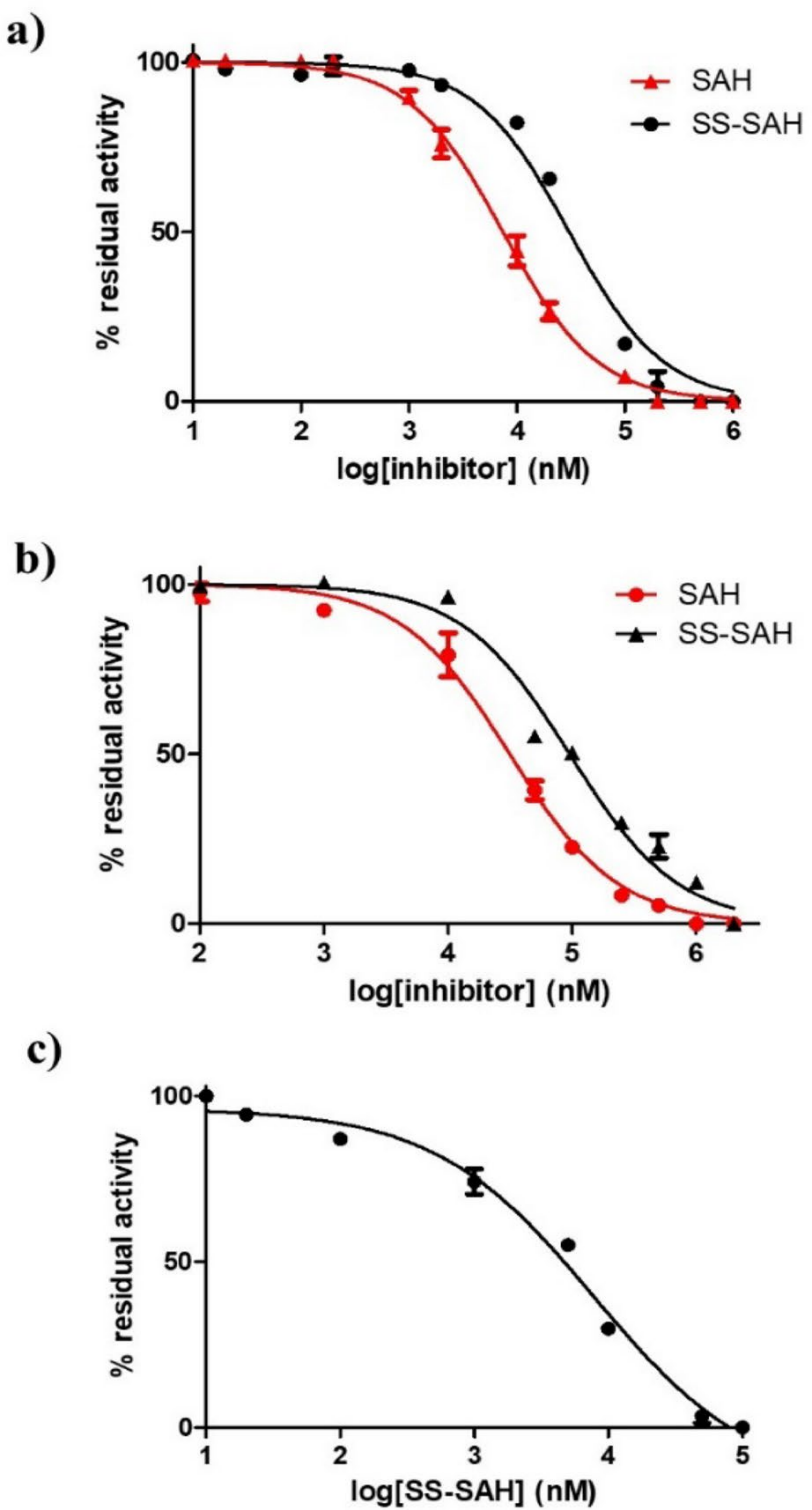
Dose-response curves for inhibition of (**a**) GLP and (**b**) G9a by SAH or **SS-SAH**, and (**c**) SETD8 by **SS-SAH**. Assay conditions: (**a**) 200 nM GLP, 5 µM histone H3K9 peptide, 20 µM SAM, (**b**) 200 nM G9a, 5 µM histone H3K9 peptide, 20 µM SAM, (**c**) 4 µM SETD8, 10 µM histone H4K20 peptide, 40 µM SAM. Assays were performed in independent replicates (*n* = 2) and all points include SEM.

MALDI-TOF MS-based assays were then carried out to determine the ability of **SS-SAH** to inhibit the activity of histone lysine methyltransferases GLP, G9a and SETD8, three biomedically important epigenetic enzymes that catalyze the trimethylation of histone H3K9 (GLP and G9a)^5^ and monomethylation of histone H4K20 (by SETD8)^34^. The redox-inactive SAH was investigated in parallel with the redox-active **SS-SAH**. In these assays we used previously reported conditions^35,36^ to compare the inhibitory activity of the two compounds (see Materials and Methods). **SS-SAH** showed to be less potent than SAH in inhibition of GLP (IC_50_ = 7.4 µM for SAH and IC_50_ = 30.4 µM for **SS-SAH**) and G9a (IC_50_ =31.4 µM for SAH and IC_50_ = 95.4 µM for **SS-SAH**). In contrast, **SS-SAH** was more potent against SETD8 (IC_50_ = 6.8 µM). SAH displayed very poor SETD8 inhibitory activity (IC_50_ > 100 µM), which initially seemed contradictory to its available crystal structures with SETD8 (Table 1, Fig. S16), however, it was indeed reported by Strelow et al. that SAH is a poor inhibitor of SETD8 when incubated with the enzyme in the presence of a protein substrate^37^. The dose-response curves for both inhibitors against GLP and G9a and the dose-response curve for **SS-SAH** against SETD8 are shown in Fig. 3.

**Table 1:** IC_50_ values (± SEM) for inhibition of KMTs by SAH and **SS-SAH**.

Compound	IC_50_ (GLP)	IC_50_ (G9a)	IC_50_ (SETD8)
**SAH**	7.4 ± 0.7 µM	31.4 ± 1.5 µM	> 100 µM
**SS-SAH**	30.4 ± 2.8 µM	95.4 ± 1.7 µM	6.8 ± 1.8 µM

Given the observed inhibition towards GLP, restrained molecular docking studies were carried out to predict the possible binding modes of **SS-SAH** and compare them to SAH. Because the structures of SAH and **SS-SAH** are very similar, it was not surprising that the docking algorithm was able to generate binding poses similar to the co-crystalized SAH molecule for both complexes. It was, however, observed in the docking of **SS-SAH** to GLP, that for the docking to properly fit the molecule inside the binding pocket, it had to reorient the amino acid moiety, causing the amine to move towards the backbone of Trp1107 (Fig. S17a). In contrast, in the docking to SETD8, the docking was able to adjust the rotatable bonds of **SS-SAH** to reestablish the positioning of the amino acid group observed in the crystal structure, thus suggesting a higher tolerance for **SS-SAH** inside the SETD8 binding pocket (Fig. S17b).

**Fig. 4:**
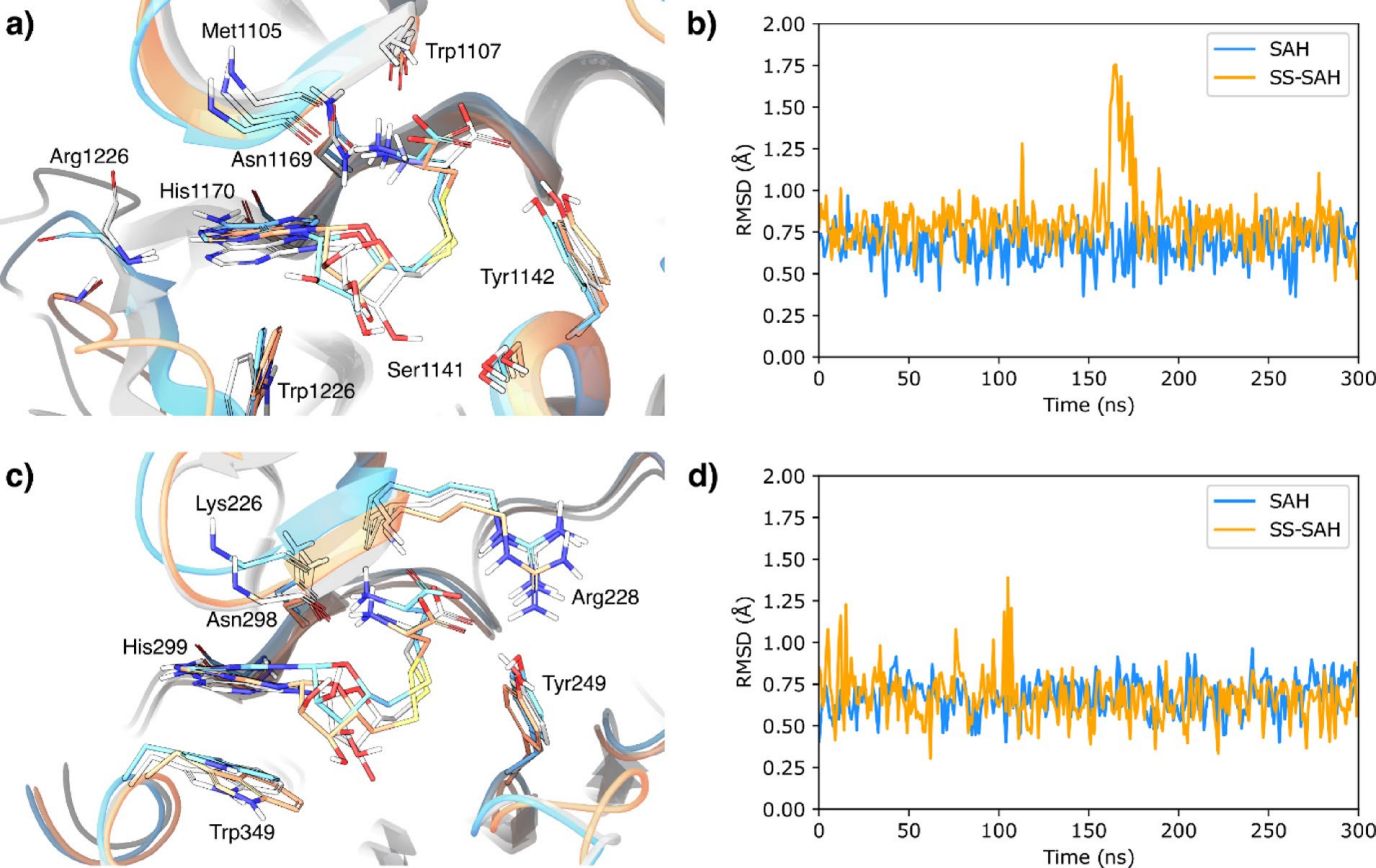
MD simulations of SAH and **SS-SAH** bound at the binding site of (**a**–**b**) GLP (PDB ID: 3HNA) and (**c**–**d**) SETD8. (**a**, **c**) Last frame of the MD simulations compared to the crystal structure. Orange: **SS-SAH** simulation, blue: SAH simulation, grey: SAH crystal structure. (**b**, **d**) RMSD of SAH/**SS-SAH** during the MD simulations. Images in panels (**a**) and (**c**) were made in Maestro from the Schrödinger Suite 2019-1 (https://www.schrodinger.com/platform/products/maestro/).

As protein flexibility is not captured in these docking experiments, we further investigated the abilities of the binding sites to accommodate **SS-SAH** and SAH using molecular dynamics (MD) simulations. For each protein and each ligand, we performed a 300 ns MD simulation. From the MD simulations it was generally observed that the binding conformation of the two molecules remained stable throughout most of the simulations of both proteins (Fig. 4). However, in the simulation of **SS-SAH** bound to GLP, a temporary jump in the RMSD was observed between 150 and 175 ns (Fig. 4b). This drastic change in RMSD was observed to coincide with a rotation around the S-S bond in **SS-SAH**, which might have caused the disturbance in the binding conformation of the entire molecule (Figs. S18-S19). A sudden change in RMSD was observed for **SS-SAH** in the simulation of **SS-SAH** bound to SETD8 at 105 ns as well, but with a smaller dihedral rotation and for a shorter interval (Fig. 4d, Figs. S18-S19). When comparing the final snapshots of all simulations, it appears that the position of the linker between the amino acid group and the sugar unit is changed notably more in the simulations of SETD8 than the simulations of GLP. This observation combined with the docking results indicate a higher tolerance for flexibility in the linker region of SAH/**SS-SAH** at the binding site of SETD8 compared to GLP, which could in part explain the higher potency experimentally observed for **SS-SAH** towards SETD8 over GLP.

**Fig. 5:**
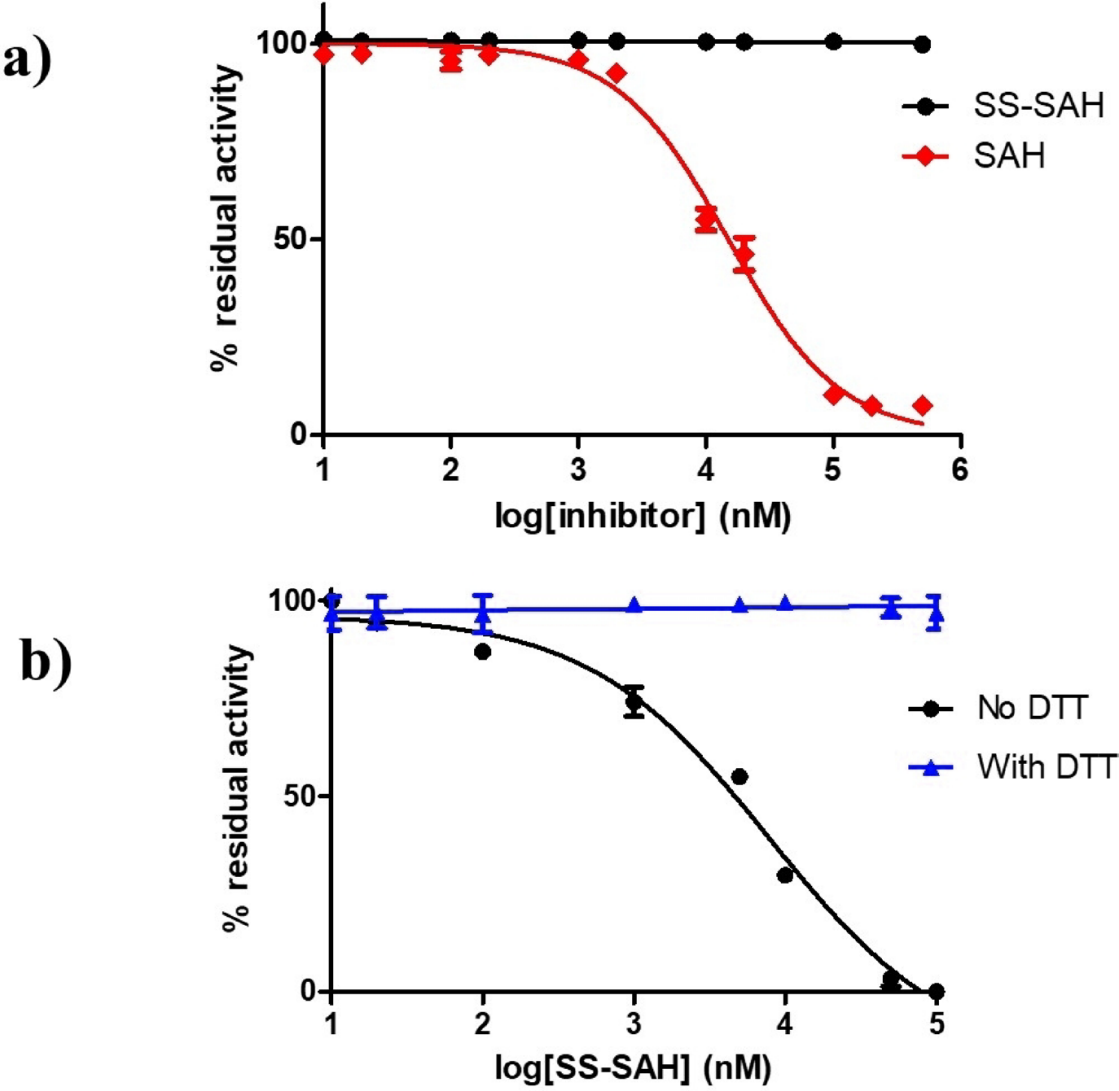
(**a**) Dose-response curves for inhibition of GLP by SAH and **SS-SAH**, in the presence of 500 µM of DTT. Assay conditions: 200 nM GLP, 5 µM histone H3K9 peptide, 20 µM SAM (**b**) Dose-response curves for inhibition of SETD8 by **SS-SAH**, in the presence and absence of 500 µM of DTT. Assay conditions: 4 µM SETD8, 10 µM histone H4K20 peptide, 40 µM SAM. Assays were performed in independent replicates (*n* = 2) and all points include SEM.

The redox lability of **SS-SAH**, and by extension its ability to be rapidly deactivated in the presence of a reductive agent, was then put to the test. In a preliminary trial, the inhibition capacity of **SS-SAH** and SAH was tested again against GLP, but this time with addition of 500 µM of DTT. SAH showed only a small increase in its IC_50_ value (14.9 µM), whereas **SS-SAH** showed a complete loss of inhibition potency, indicating that the reducing conditions have a dramatic effect on the inhibitory potency of **SS-SAH** due to the cleavage of the S-S bond and formation of inactive cysteine and mercaptoadenosine **3** (Fig. 5a). Furthermore, **SS-SAH** showed a loss of potency against SETD8 under the same conditions and addition of 500 µM of DTT (Fig. 5b).

**Fig. 6:**
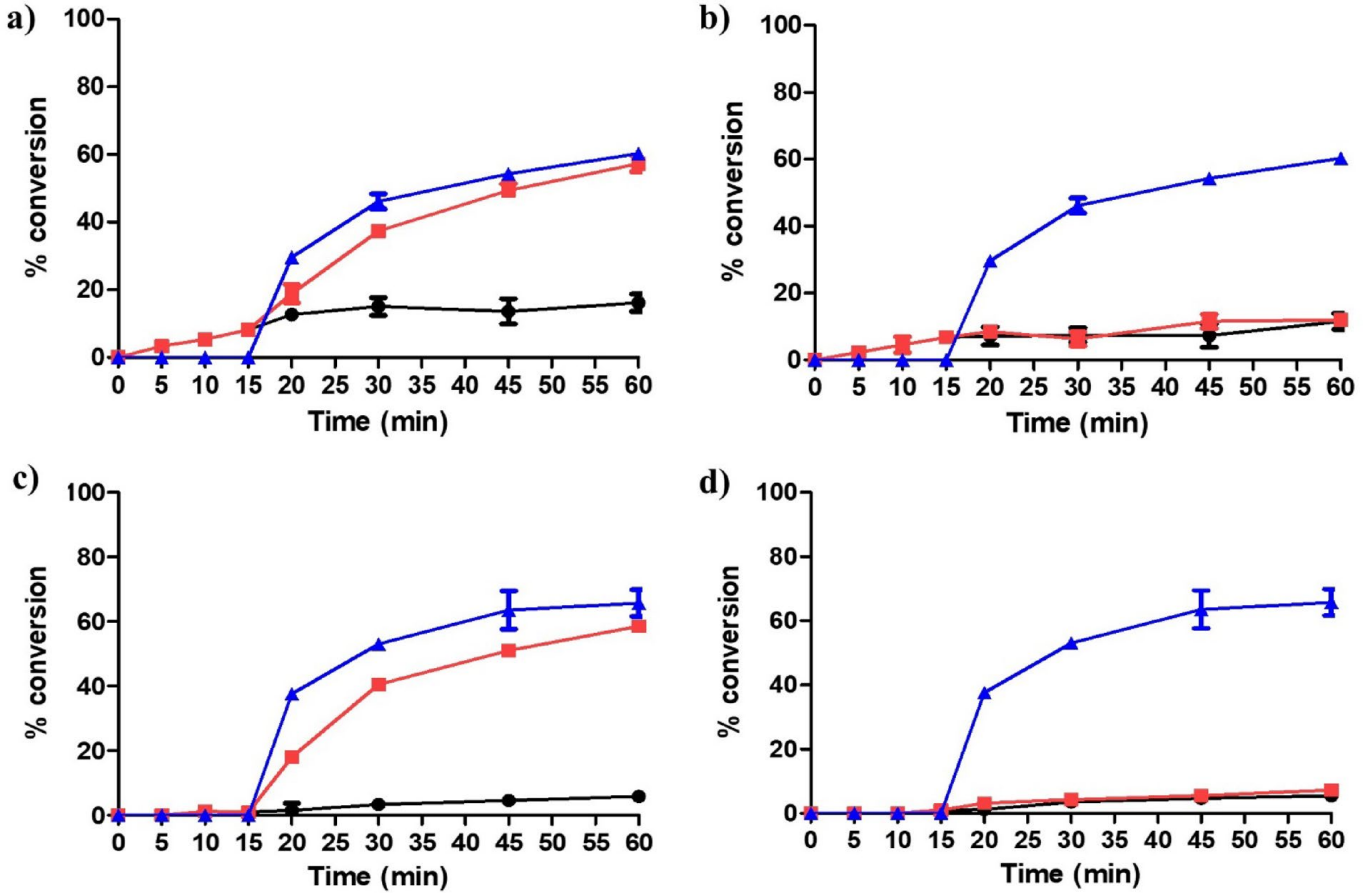
Methylation of histone H3K9 peptide (5 µM) by GLP (200 nM) over time in the presence of **SS-SAH** or SAH and addition of DTT. (**a**) **SS-SAH** (100 µΜ) and DTT (500 µΜ). (**b**) SAH (100 µΜ) and DTT (500 µΜ). (**c**) **SS-SAH** (500 µΜ) and DTT (2.5 mM). (**d**) SAH (500 µΜ) and DTT (2.5 mM). Black: Conversion in presence of inhibitor, Red: Conversion in presence of inhibitor and addition of DTT at 15 min, Blue: Conversion in the absence of inhibitor, initiated with addition of SAM (20 µM) at 15 min. The % conversion is defined as the percentage of all possible methylations completed, with 100% conversion being the entire amount of H3K9 being methylated 3 times. Assays were performed in independent replicates (*n* = 2) and all points include SEM.

Following these results, the ability of GLP to recover its activity over time, with the reduction of **SS-SAH** in the presence of DTT was tested under different conditions. This trend was also compared to incubating the enzyme with redox-inactive SAH instead of **SS-SAH** and observing the GLP activity following addition of DTT. Thus, GLP was chosen, as both compounds show substantial inhibition with this enzyme. First, the experiment was carried out under the standard inhibition assay conditions, in the presence of 100 or 500 µM of each inhibitor, where 500 µM or 2.5 mM (i.e., 5 equiv) of DTT, respectively, were added after 15 min of reaction time. The percentage of methylation of 13-mer H3K9 peptide was measured in intervals from 0 to 60 min (Figs. 6, S20-S23). This was compared to the conversion (%) of H3K9 methylation in the presence of the inhibitors without adding DTT, as well as the conversion (%) in the presence of DTT and in the absence of inhibitors, as a positive control. Here, % conversion is defined as the percentage of all possible methylations completed, with 100% conversion being the entire amount of H3K9 being methylated 3 times. It can be observed that addition of DTT to **SS-SAH** leads to significant recovery of enzymatic activity of GLP when compared to the control in the absence of added DTT during 60 min incubation time (Figs. 6a, c, S20, S22). Specifically, GLP (200 nM) can be observed to slowly regain activity up to 95% at 60 min by the addition of 500 µM DTT to 100 µM of **SS-SAH** (Figs. 6a, S20). In the case of adding 2.5 mM of DTT to 500 µM of **SS-SAH**, 89% activity recovery can be observed within 45 min of the addition of DTT (Figs. 6c, S22). In the case of SAH, adding 500 µM of DTT to 100 µM of SAH (Figs. 6b, S21) or 2.5 mM of DTT to 500 µM of SAH (Figs. 6d, S22) have no apparent effect in its inhibitory activity against GLP, demonstrating its redox-inactive properties. The same experiment was repeated with 100 µM inhibitor, 500 µM of DTT and 100 or 500 nM of GLP (Figs. S24-S28). In the case of 100 nM of GLP, nearly full activity recovery was observed immediately after DTT addition, however, a significant drop in the overall activity can be observed, due to decreased enzyme concentration. At 500 nM of GLP, on the other hand, the effect of **SS-SAH** reduction is not very pronounced. Despite up to 95% recovery being observed at 60 min, GLP is relatively poorly inhibited under these conditions by both SAH and **SS-SAH**. Finally, this experiment was repeated with keeping GLP concentration at 200 nM, increasing the concentration of the inhibitors to 500 µΜ, but reducing the DTT concentration to 1 mM. The aim was to observe the recovery of enzymatic activity with decreased DTT. In this case, a high GLP activity recovery was observed (86%) (Figs. S29-S31).

## Conclusion

**SS-SAH**, a novel redox-labile analog of SAH, was designed and synthesized in a facile manner. Inhibition assays demonstrated that SAH and **SS-SAH** act as inhibitors of biomedicinally important histone lysine methyltransferases GLP and G9a, as well as SETD8 in the case of **SS-SAH**. Molecular docking studies combined with molecular dynamics simulations provided an insight into binding modes and dynamics of **SS-SAH** and SAH in complex with GLP. These results indicate that **SS-SAH** fits in the GLP active site, albeit less favorably compared to SAH. **SS-SAH** showed higher IC_50_ values than SAH for inhibition of GLP and G9a, possibly limiting the current scope of KMTs it can be used for. However, the observed changes in inhibitory activity and binding mode provide important knowledge about their binding pose and potency, especially given that these changes were quite significant despite very subtle modifications to SAH. Additionally, it should be acknowledged that the current work is limited to a small set of KMTs. Further research on other classes of methyltransferases is needed and could provide even more interesting insights into their inhibition by structures such as **SS-SAH**. Furthermore, due to its redox-labile properties, **SS-SAH** was able to effectively deactivate the human lysine methyltransferase GLP and gradually activate it again, by adjusting the redox conditions per demand. This approach provides a unique advantage when compared with common reversible inhibitors, including redox inactive SAH as a pan-inhibitor of KMTs, which was found to maintain its stable inhibitory potency. The disulfide bond in **SS-SAH** does likely pose some limitations; in a cellular or in vivo context, endogenous thiols such as glutathione can reduce the compound, decreasing its inhibitory potency, while a similar effect can occur in in vitro environments requiring high concentrations of DTT. Nevertheless, flexibility and immediate control over this structure offers useful applications for in vitro studies of other biomedically important methyltransferases of current therapeutic interest. Based on the structure of **SS-SAH**, there is potential of further modifications, to increase potency and selectivity against methyltransferases important for human health and disease. We hope that the redox-labile properties of **SS-SAH** and its use for redox-dependent deactivation/reactivation of GLP activity will inspire design and development of inhibitors of other biomedically important enzymes.

## Materials and methods

### General experimental procedures

^1^H and ^13^C NMR spectra were recorded using a Bruker Avance III 400 MHz NMR spectrometer and a JEOL JNM-ECZR 500 MHz NMR spectrometer. High-resolution mass spectra (HRMS) were recorded on a Bruker Daltonics-micrOTOF-Q II-ESI-Qq-TOF mass spectrometer. Chemical shifts are reported in parts per million (ppm δ) referenced to the residual 1H resonance and the residual 13C resonance of the deuterated solvent. Splitting patterns are designated as follows: s, singlet; d, doublet; dd, doublet of doublets; ddd, doublet of doublet of doublets; t, triplet; td, triplet of doublet; q, quartet; and m, multiplet. Coupling constants (J) are reported in hertz (Hz). MALDI-TOF spectra were measured in a UltrafleXtreme-II tandem mass spectrometer (Bruker). Analytical RP-HPLC employed a Gemini 5 mm C18 110 LC column (Phenomenex) at a flow rate of 1 mL/min with a gradient of H_2_O + 0.1% TFA and ACN + 0.1% TFA from 1 (v/v) for 1 min to 100% ACN + 0.1% (v/v) TFA at 11 to 15 min and back to 10 from 16 to 20 min. Analytical spectra were monitored at 254 nm. All reagents and solvents were purchased from commercial sources and used without further purification.

### Synthetic procedures

#### Synthesis of **1**^25^

To an ice-cold solution of triphenylphosphine (1.90 g, 7.2 mmol) in dry THF (10 mL), diisopropyl azodicarboxylate (1.42 mL, 7.2 mmol) was added dropwise. After stirring for 30 min, 2′,3′-O-isopropylideneadenosine (1.00 g, 3.26 mmol) was added, and stirring was continued for 10 min. To the resulting suspension, a solution of thioacetic acid (0.53 mL, 7.2 mmol) in dry THF (2 mL) was added dropwise and the stirring was continued for 1 h at 0 °C. After consumption of the starting material, indicated by TLC, the solvent was removed under reduced pressure and the resulting residue was purified by flash chromatography (THF in DCM 20% then MeOH in DCM 5%), to yield 833 mg of **1** (70%). ^1^H NMR (400 MHz, CDCl_3_) δ 8.37 (s, 1H), 7.90 (s, 1H), 6.07 (d, J= 2.1 Hz, 1H), 5.69 (s, 2 H), 5.52 (dd, J = 6.3, 2.1 Hz, 1H), 4.98 (dd, J = 6.3, 3.1 Hz, 1H), 4.35 (td, J = 6.9, 3.1 Hz, 1H), 3.34–3.14 (m, 2 H), 2.35 (s, 3 H), 1.60 (s, 3 H), 1.39 (s, 3 H). ^13^C NMR (101 MHz, CDCl_3_) δ 194.5, 155.6, 153.2, 149.3, 140.1, 120.4, 114.6, 91.0, 86.2, 84.2, 83.8, 31.3, 30.6, 27.1, 25.4. ESI-MS: calculated for C_15_H_20_N_5_O_4_S [M + H]^+^: 366.1231; found: 366.1213.

#### Synthesis of **2**^25^

Compound **1** (120 mg, 0.32 mmol) was dissolved in a formic acid/water mixture 1:1 (4 mL), and the mixture was stirred at room temperature for 48 h. The solvent was concentrated under reduced pressure and then co-evaporated 3x with toluene. The resulting solid was dried under the vacuum line overnight to yield **2** in quantitative yield (106 mg). ^1^H NMR (500 MHz, DMSO-D_6_) δ 8.38 (s, 1H), 8.18 (s, 1H), 7.64 (s, 2 H), 5.85 (d, J = 5.8 Hz, 1H), 4.73 (t, J = 5.4 Hz, 1H), 4.06 (dd, J = 5.0, 3.6 Hz, 1H), 3.89 (ddd, J = 7.4, 5.7, 3.6 Hz, 1H), 3.34–3.10 (m, 2 H), 2.29 (s, 3 H). ^13^C NMR (126 MHz, DMSO-D_6_) δ 195.4, 170.7, 155.6, 151.9, 149.8, 141.0, 119.7, 88.0, 83.5, 73.2, 73.1, 40.5, 40.4, 40.3, 40.2, 40.2, 40.1, 40.1, 39.8, 39.7, 39.5, 31.7, 31.0. ESI-MS: calculated for C_12_H_16_N_5_O_4_S [M + H]^+^: 326.0918; found: 326.0920.

#### Synthesis of **3**^25^

Before the reaction, 7 mL of a solution of aqueous ammonia 7 N in methanol was degassed through 5 freeze/thaw cycles under vacuum. The nucleoside **2** (50 mg, 0.16 mmol) was dissolved in the methanolic ammonia solution under argon, and the reaction mixture was stirred at 0 °C. After 30 min, methanol was evaporated under reduced pressure, the remaining aqueous reaction mixture was frozen with liquid nitrogen, and the solvent was removed by lyophilization to yield **3** (45 mg), which was used in the next step without further purification. ^1^H NMR (400 MHz, DMSO-D_6_) δ 8.36 (s, 1H), 8.16 (s, 1H), 7.31 (s, 2 H), 5.94–5.85 (m, 1H), 4.82–4.74 (m, 1H), 4.18 (dd, J = 5.1, 3.2 Hz, 1H), 3.97 (td, J = 6.2, 3.2 Hz, 1H), 2.83 (ddd, J = 44.2, 13.7, 6.2 Hz, 2 H), 2.51 (m, 2 H). ^13^C NMR (101 MHz, DMSO-D_6_) δ 156.07, 152.60, 149.45, 139.95, 119.21, 87.31, 85.39, 72.58, 71.89, 26.48. ESI-MS: calculated for C_10_H_14_N_5_O_3_S [M + H]^+^: 284.0812; found: 284.0813.

#### Synthesis of **4**^29^

L-Cysteine (1.21 g, 10 mmol) was dissolved in 10 mL of 3 N HCl (aq) and cooled to 0 °C in an ice−water bath. While stirring the solution, sodium nitrite (690 mg, 10 mmol) dissolved in 5 mL water was added dropwise, and the deep red solution was stirred for 40 min in ambient atmosphere. Sodium methane sulfinate (2.04 g, 20 mmol) dissolved in 5 mL of water was added rapidly and the solution was stirred at 0 °C for an additional 3.5 h. Then, more sodium methane sulfinate (510 mg, 5 mmol) dissolved in 5 mL of water was added rapidly. The solution was stirred for an additional 30 min, until disappearance of the red color. The resulting suspension was filtered and the solid was washed with 100 mL of ice-cold water and 100 ml of diethyl ether, followed by drying under high vacuum to afford **4** as a white solid (1.03 g, 52%). ^1^H NMR (500 MHz, D_2_O) δ 4.37 (dd, J = 6.8, 4.6 Hz, 1H), 3.78–3.54 (m, 2 H), 3.40 (s, 3 H). ^13^C NMR (126 MHz, D_2_O) δ 169.29, 52.53, 49.71, 34.68. ESI-MS: calculated for C_4_H_10_NO_4_S_2_ [M + H]^+^: 200.0046; found: 200.0056.

#### Synthesis of **SS-SAH**

20 mg of **4** (0.1 mmol) were suspended in 1 ml of absolute MeOH under argon. Then 2 eq of triethylamine were added and the stirring solution was cooled to 0 °C. Then, a solution of the crude **3** (45 mg) in absolute MeOH was added dropwise and the mixture was slowly warmed to room temperature and stirred for 6 h. The solvent was then removed under reduced pressure and the resulting crude solid was purified through reverse-phase preparative HPLC (RP-HPLC) using a gradient of H_2_O + 0.1% TFA and ACN + 0.1% TFA from 0 to 7% ACN in 15 min to 70% ACN in 30 min at 10 ml/min^38^ and a Gemini 10 μm NX-C18 110 Å LC column (Phenomenex, Torrance, CA, USA). The collected fractions were combined and lyophilized to yield 25 mg of **SS-SAH** (0.06 mmol, 62%). ^1^H NMR (500 MHz, D_2_O) δ 8.38 (s, 1H), 8.31 (s, 1H), 6.03 (d, J = 5.3 Hz, 1H), 4.82–4.78 (m, 1H), 4.39–4.31 (m, 2 H), 4.00 (dd, J = 8.1, 4.1 Hz, 1H), 3.24–3.00 (m, 4 H). ^13^C NMR (126 MHz, D_2_O) δ 172.16, 150.26, 148.44, 144.85, 142.99, 119.09, 88.48, 83.26, 73.53, 72.37, 52.92, 40.01, 37.92. ESI-MS: calculated for C_13_H_19_N_6_O_5_S_2_ [M + H]^+^: 403.0853; found: 403.0833.

### GLP/G9a expression and purification

The methyltransferases (GLP and G9a) were expressed and purified as previously described^39^. The wild-type enzymes were recombinantly expressed in *E.coli* Rosetta BL21 (DE3)pLysS cells and the bacterial cells were grown in LB medium containing 100 mg/mL kanamycin at 37 °C using an incubator until their OD600 reached 0.5–0.6 (approximately 3–4 h). Protein expression was induced with isopropyl-beta-D-thiogalactopyranoside (IPTG) and the cells were then incubated at 16 °C overnight. Cells were harvested by centrifugation at 4000 rpm, at 4 °C for 15 min, and the cell pellets were resuspended in lysis buffer (Complete Protease inhibitor cocktail tablet, Roche). Cell lysis was performed with sonicator at an amplitude for 20 s (8 times) with 90 s intervals, while keeping the cells cooled at an ice water bath. Cell debris was removed by centrifugation and then the expressed proteins were loaded onto a Ni-NTA affinity column. The lysis buffer was washed, and bound proteins were eluted using a linear gradient concentration of imidazole. All proteins were then subjected to size exclusion chromatography (SEC) and the purified proteins were concentrated with Amicon Ultra Centrifugal Filter Units (Millipore) with suitable molecular weight cut-offs (10 kD). The protein concentrations were determined using the Nanodrop DeNovix DS-11 spectrophotometer and the purity was monitored by SDS-PAGE on a 4–15% gradient polyacrylamide gel.

### SETD8 expression and purification

The expression and purification of SETD8 (residues 186–352) was carried out as previously described^40^. Briefly, His-tagged SETD8 was expressed in *E. coli* Rosetta BL21 (DE3)pLysS cells in the presence of 1.0 mM IPTG (final concentration) and cultured overnight at 16 °C. Harvested cells were re-suspended into 20 mM Tris pH 7.8, 500 mM NaCl, 5 mM imidazole, 5% glycerol, and 5 mM β-mercaptoethanol, and subsequently lysed by sonication. The cell lysate was then incubated with Ni-NTA beads, and the beads were then washed with 20 mM Tris pH 7.8, 500 mM NaCl, 20 mM imidazole and 5% glycerol, and the protein was subsequently eluted with 20 mM Tris pH 7.8, 500 mM NaCl, 250 mM imidazole and 5% glycerol. The eluted protein was concentrated using a spinfilter device (Amicon, 3.5 MWCO) and was further purified by gel filtration chromatography, using a Superdex 75 column and 20 mM Tris pH 7.5, 100 mM NaCl and 10 mM β-mercaptoethanol at 0.5 ml min^− 1^ flow speed. Fractions with correct molecular weight and best purity based on SDS-PAGE were pooled and concentrated, aliquoted, snap-frozen, and stored at − 80 °C.

### Histone peptide synthesis and purification

The H3 and H4 peptides were synthesized as previously described^41^. Briefly, it was synthesized using microwave assisted solid-state peptide synthesis (SPPS) on a Rink-amide resin, with a Liberty Blue peptide synthesizer (CEM corporation, Matthews, NC, USA). Amino acid couplings were carried out with an equivalent ratio of [5]: [5]: [7.5] of [Fmoc-NH amino acid]: [DIC]: [Oxyma Pure] at 75 °C for 2 min and the Fmoc group was removed with 20% piperidine in DMF. The peptide was then cleaved from the resin using 0.25% TIPS, 0.25% H_2_O, 99.5% TFA for 4 h. The crude peptide was precipitated by addition of cold Et_2_O and after suspension it was centrifuged for 5 min at 5000 rpm in an Eppendorf 5804R centrifuge (Eppendorf, Hamburg, Germany), after which the supernatant was removed. Washing of the remaining solid with cold Et_2_O and centrifugation were repeated twice more. The crude peptide was dissolved in a mixture of ACN in H_2_O and purified using preparative reverse-phase HPLC (RP-HPLC) using a gradient of H_2_O + 0.1% TFA and ACN + 0.1% TFA from 5% ACN to 30% ACN over 30 min at 4 ml/min using a Gemini 10 μm NX-C18 110Å LC column (Phenomenex, Torrance, CA, USA). Analytical RP-HPLC was carried out on a Gemini 5 μm C18 110Å LC column (Phenomenex) at a flow rate of 1 mL/min. Analytical injections were monitored at 215 nm. The mass of the peptide was confirmed by mixing with a 1:2 with α-Cyano-4-hydroxycinnamic acid (α-CHCA) matrix and loaded onto an MTP 384 polished steel target to be analyzed by MALDI-TOF.

### **SS-SAH** degradation by DTT and GSH

**SS-SAH** (100 µM) was incubated with either 1 mM, 500 µM or 200 µM of reducing agent DTT or 1 mM of GSH for 60 min in 50 mM Tris-HCl buffer, pH = 8.0. The degradation of **SS-SAH** was monitored by analytical HPLC at 254 nm after between 0 min and 60 min. The analytical RP-HPLC employed a Gemini 5 mm C18 110 LC column (Phenomenex) at a flow rate of 1 mL/min with a gradient of H_2_O + 0.1% TFA and ACN + 0.1% TFA from 1 (v/v) for 1 min to 100% ACN + 0.1% (v/v) TFA at 11 to 15 min and back to 10 from 16 to 20 min. LC-MS analyses used an ELUTE HPLC system (Bruker Daltonics, Germany) connected to a Dual Absorbance Detector (Bruker Daltonics, Germany) and MS Impact II electrospray ionization quadrupole time-of-flight MS from Bruker Daltonics (Bremen, Germany). The mobile phase consisted of ultra-pure water (A) and ACN (B) and containing 0.1% FA. A reversed phase column (Bio-AQ C18 100 mm, 2.1 mm ID, Bruker, Germany) was applied and maintained at 37 °C. The flow was constant at 0.4 mL/min with an elution starting with 90% A and 10% B changing to 0% A and 100% B over 11 min. maintained for 4 min. Changing to 90% A and 10% B over 1 min. maintained for 4 min. Total runtime of 20 min. Samples of 5 µL, maintained at 20 °C, were injected and detected at 254 nm.

### MALDI-TOF inhibition assays

For GLP/G9a: The mono-, di- and tri-methylation of a 13-mer histone H3K9 peptide (sequence: ARTKQTARKSTGG) was performed. First, the enzyme (GLP or G9a, at 200 nM final concentration) and **SS-SAH** at a range of final concentrations of 10 nM–1 mM (from a stock in MilliQ-water) were preincubated for 5 min in reaction buffer (50 mM TRIS pH 8.0). After preincubation, histone peptide (final concentration 5 µM) and SAM (final concentration 20 µM) were added, at a final volume of 20 µL. The mixture was incubated for 30 min at 37 °C, after which it was quenched by the addition of 20 µL of 10% TFA in MilliQ-water. For MALDI-TOF MS analysis, a 2 µL aliquot of the quenched reaction mixture was mixed with 2 µL of α-CHCA matrix and from this 2 µL was loaded onto an MTP 384 polished steel target to be analyzed by MALDI-TOF. The total peak area of each methylation state, with all isotopes and adducts, were used for determination of enzymatic activity in the presence of **SS-SAH** relative to the activity in the absence of **SS-SAH**. The assay was performed in two independent replicates, and each point is presented as the mean of the replicates, including the standard error.

### For SETD8

The mono-methylation of a 15-mer histone H4K20 peptide (sequence: GGAKRHRKVLRDNIQ) was performed as follows: First, the enzyme (4 μM final concentration) and the inhibitors at a range of final concentrations of 50 nM – 2 mM (from MilliQ water stock) were preincubated for 5 min in reaction buffer (50 mM TRIS pH 8.0). After preincubation, histone peptide (final concentration 10 µM) and SAM (final concentration 40 µM) were added, at a final volume of 20 µL. The mixture was incubated for 1 h at 37 °C, after which it was quenched by the addition of 20 µL of 10% TFA in MilliQ-water. For MALDI-TOF MS analysis, a 2 µL aliquot of the quenched reaction mixture was mixed with 2 µL of α-CHCA matrix and from this 2 µL was loaded onto an MTP 384 polished steel target to be analyzed by MALDI-TOF. The total peak area of each methylation state, with all isotopes and adducts, were used for determination of enzymatic activity in the presence of inhibitors relative to the activity in their absence. The assay was performed in two independent replicates, and each point is presented as the mean of the replicates, including the standard error.

### **SS-SAH** deactivation by DTT

GLP and **SS-SAH** or SAH at the appropriate concentrations were preincubated for 5 min in reaction buffer (50 mM TRIS pH 8.0). After preincubation, histone peptide (final concentration 5 µM) and SAM (final concentration 20 µM) were added, at a final volume of 80 µL. The mixture was incubated for 60 min at 37 °C, but at 15 min, the solutions were divided in half, and one half DTT was added at the desired final concentration. During the experiment, 2 µL aliquots were taken after 5, 10, 15, 20, 30, 45 and finally at 60 min, after which it was quenched by the addition of 10% TFA in MilliQ water at a 1:1 volume to the reaction volume. Additionally, a positive control was run without inhibitor, where the first 15 min, the enzyme was simply incubated with the peptide and the reaction was initiated at 15 min by addition of SAM, followed by addition of DTT. For MALDI-TOF MS analysis, a 2 µL aliquot of the quenched reaction mixture was mixed with 2 µL of α-CHCA matrix and from this 2 µL was loaded onto an MTP 384 polished steel target to be analyzed by MALDI-TOF. The total peak area of each methylation state, with all isotopes and adducts, were used for determination of enzymatic activity in the presence of **SS-SAH** relative to the activity in the absence of **SS-SAH**. The assay was performed in two independent replicates, and each point is presented as the mean of the replicates, including the standard error.

### Molecular docking

The crystal structures of GLP (PDB ID: 3HNA) and SETD8 (PDB ID: 1ZKK) were imported into the Maestro module available with Schrödinger package^42^. All water molecules were deleted from both structures, while Chain A with associated ligands was extracted for further preparation. The proteins were prepared using the Protein Preparation Wizard available in Maestro. The preparation tool was used to add hydrogen atoms, determine bond orders, and determine protonation states. Protonation states were calculated using the PROPKA implementation^43^. A restrained minimization of the system was finally performed using the OPLS4 force field. Ligands were prepared using LigPrep. Restrained docking simulations were performed using extra precision (XP) Glide, with restraints on the nucleotide and sugar heavy atoms^44,45^. Poses with maximum common scaffold RMSD > 2 Å to restrained atoms were excluded.

### MD simulations

For the starting conformation of the MD simulations, the prepared structures of GLP and SETD8 with SAH bound, also used for docking, were exported. Two simulation series were performed for each of the proteins: one with the protein bound to SAH and one with the protein bound to **SS-SAH**. GAFF2 parameters for both simulations were determined using Antechamber with the AM1-BCC charge method^46–48^. The systems were set up using tleap, where the protein was solvated in a2 TIP3P^49^ water box with a 0.150 M NaCl concentration and a 12 Å buffer distance. Simulations were performed using Amber18^46–48^ with a 2 fs timestep, and with GAFF2 parameters for SAH and **SS-SAH**, while the ff14SB force field was used for the proteins. Previously determined methyl-lysine parameters were used for this residue^50^. The simulation series were performed after a short 1000 step minimization, by first heating the system to 300 K for 50 ps. The Berendsen barostat was then applied and an additional 550 ps simulation was performed to equilibrate the system, before the final 300 ns were simulated and used for analysis.

## Supplementary Information

Below is the link to the electronic supplementary material.

Supplementary Material 1

## Data Availability

All data generated or analysed during this study are included in this published article and its supplementary information files.
