# Supplementary material for: Inhibition of human histone lysine methyltransferases by a redox-labile S-adenosyl-L-homocysteine analog
Source: Sci Rep. 2026 May 13;16:19173. doi: 10.1038/s41598-026-52984-9 (PMC13282376; doi:10.1038/s41598-026-52984-9)
Supplement: Supplementary file 1 — Supplementary Material 1 [file 41598_2026_52984_MOESM1_ESM.pdf]

Supplementary Information

Inhibition of human histone lysine methyltransferases by a redox-labile S-adenosyl-L-homocysteine analog

Faidra Voukia, Laust Moesgaard, Jacob Kongsted, and Jasmin Mecinović*

Department of Physics, Chemistry and Pharmacy, University of Southern Denmark, Campusvej 55, 5230 Odense (Denmark)

* Email: mecinovic@sdu.dk

Contents

Characterization data of synthesized compounds.. 3
Degradation of SS-SAH in the presence of DTT and GSH.. 9
Inhibition of SETD8 by SAH 16
Docking and molecular dynamics.. 17
Percentages of histone H3K9 methylation states during deactivation of SS-SAH with DTT........ 18

Characterization data of synthesized compounds

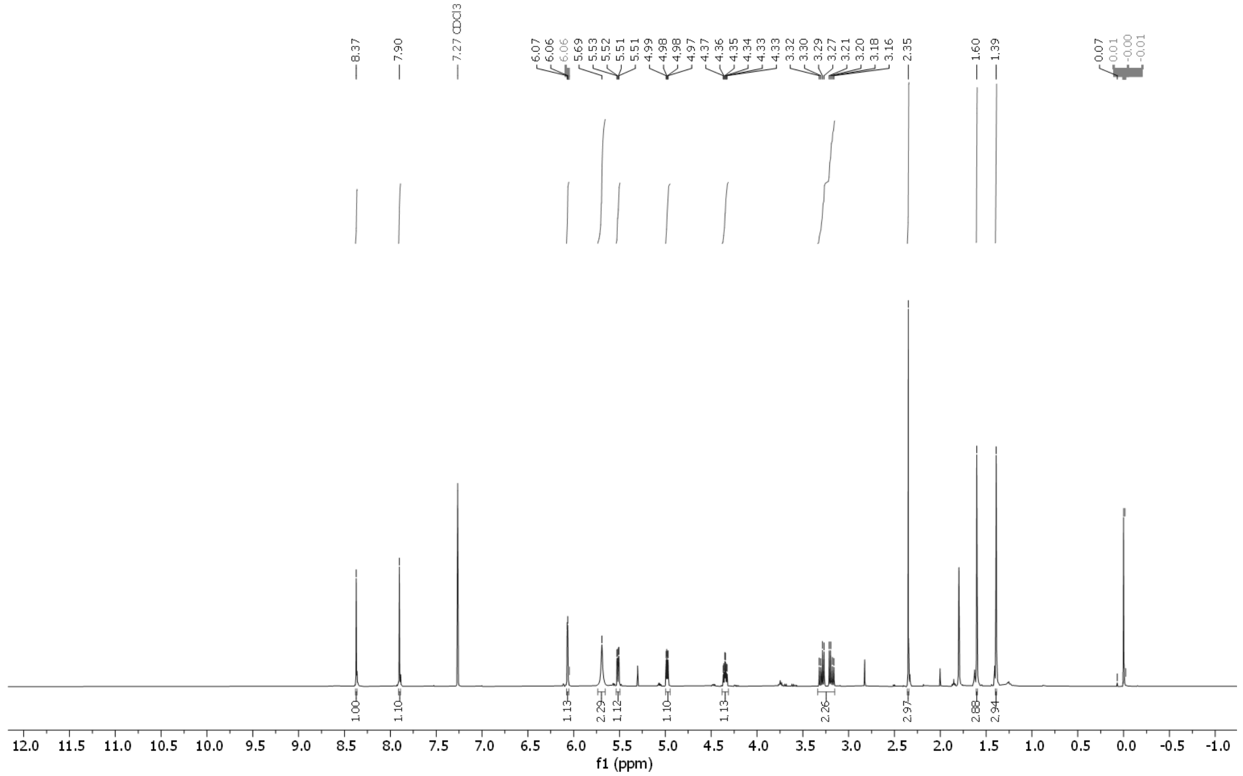

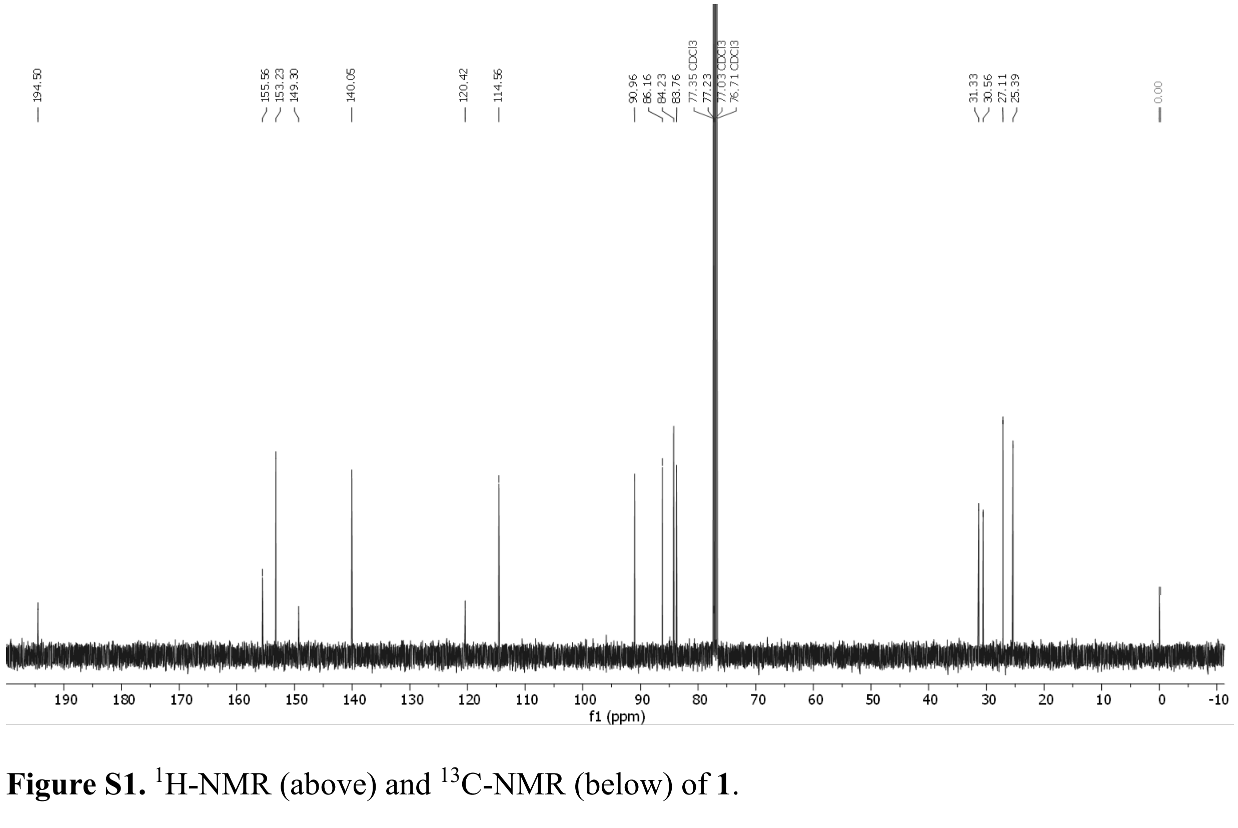

Figure S1. ^{1}H-NMR (above) and ^{13}C-NMR (below) of **1**.

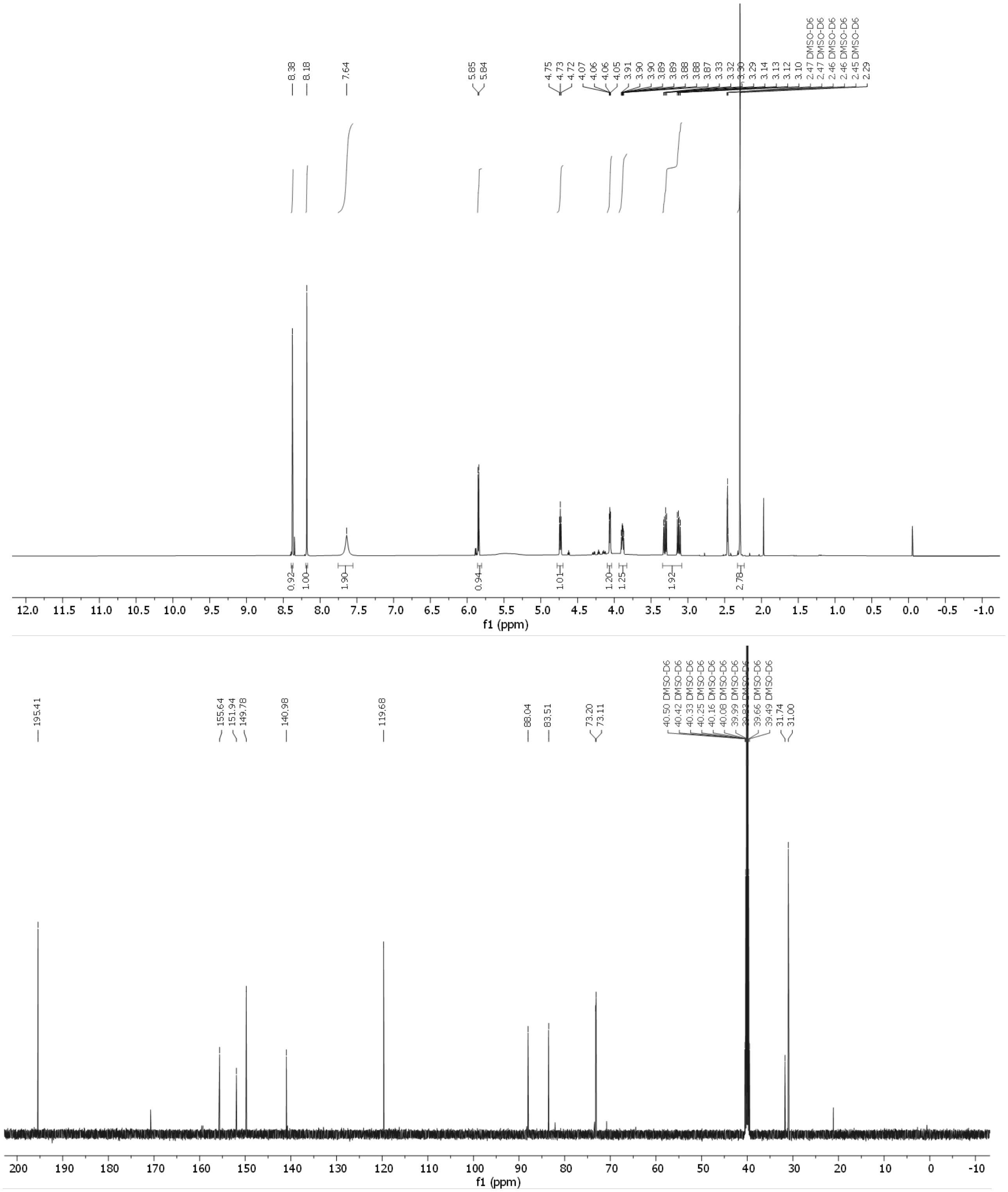

Figure S2. ^{1}H-NMR (above) and ^{13}C-NMR (below) of **2**.

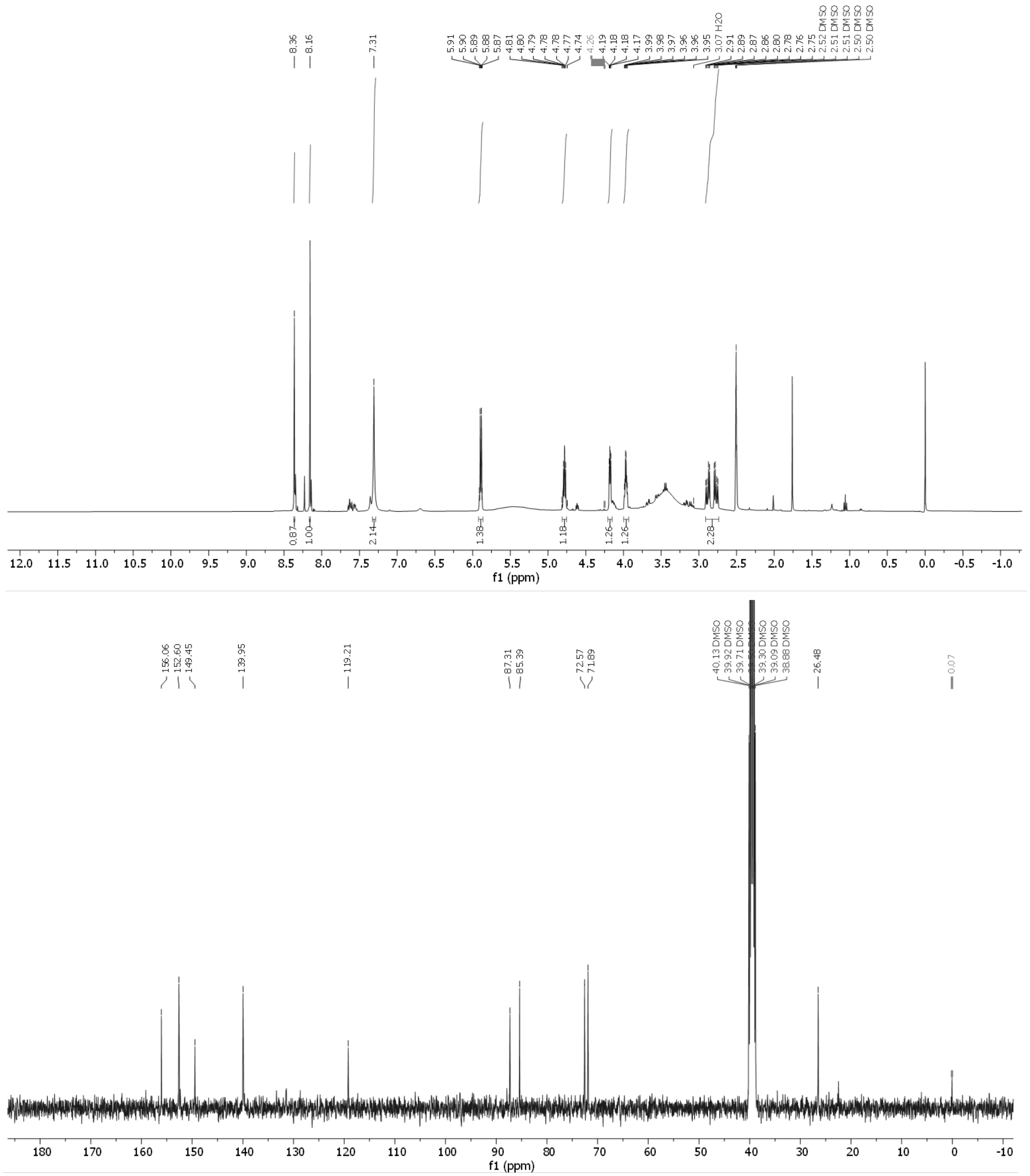

Figure S3. ^{1}H-NMR (above) and ^{13}C-NMR (below) of **3**.

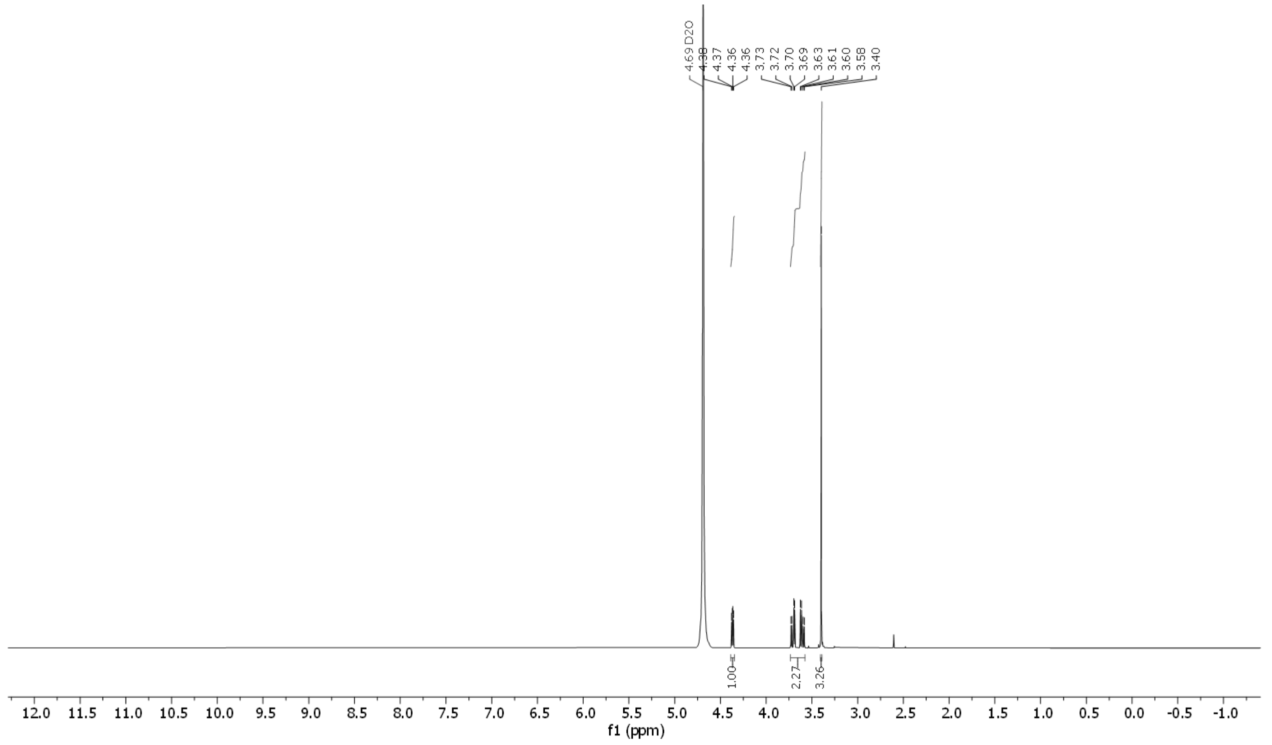

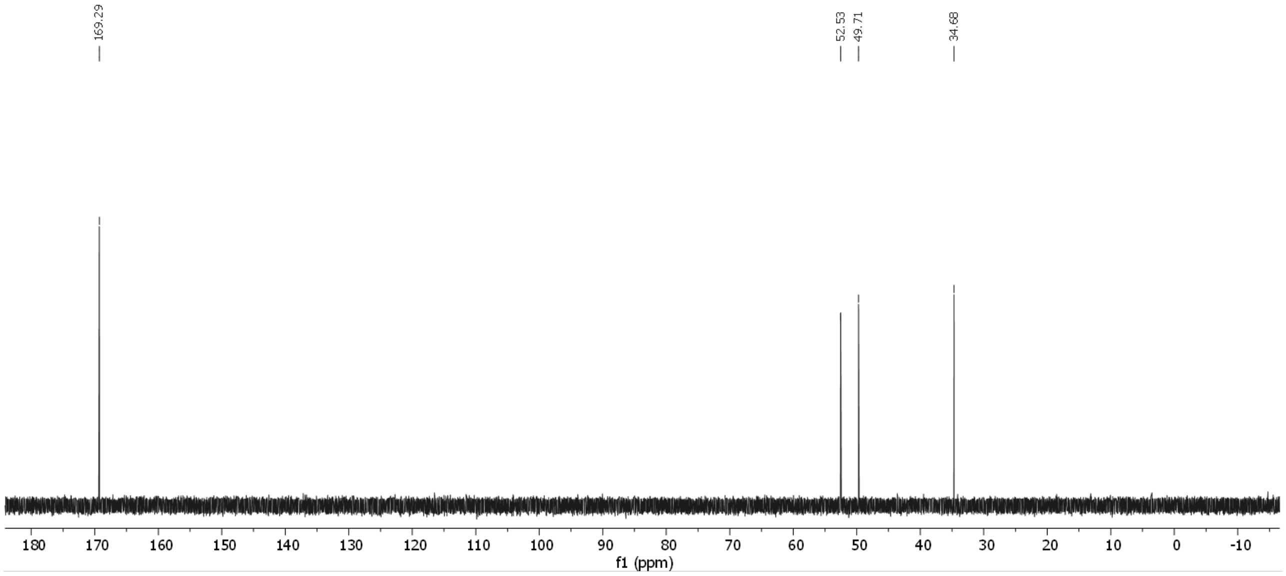

Figure S4. ^{1}H-NMR (above) and ^{13}C-NMR (below) of **4**.

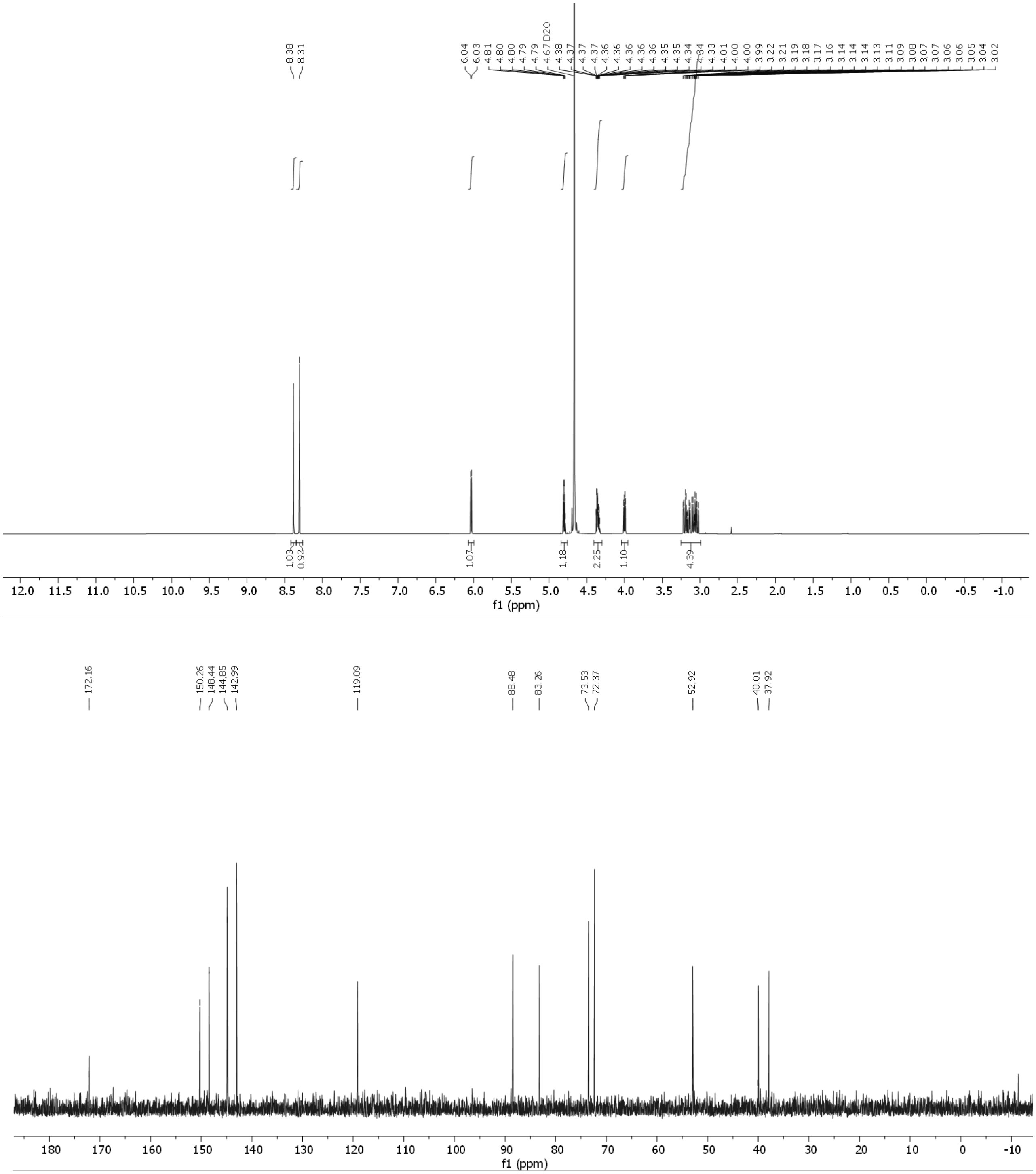

Figure S5. ^{1}H-NMR (above) and ^{13}C-NMR (below) of **SS-SAH**.

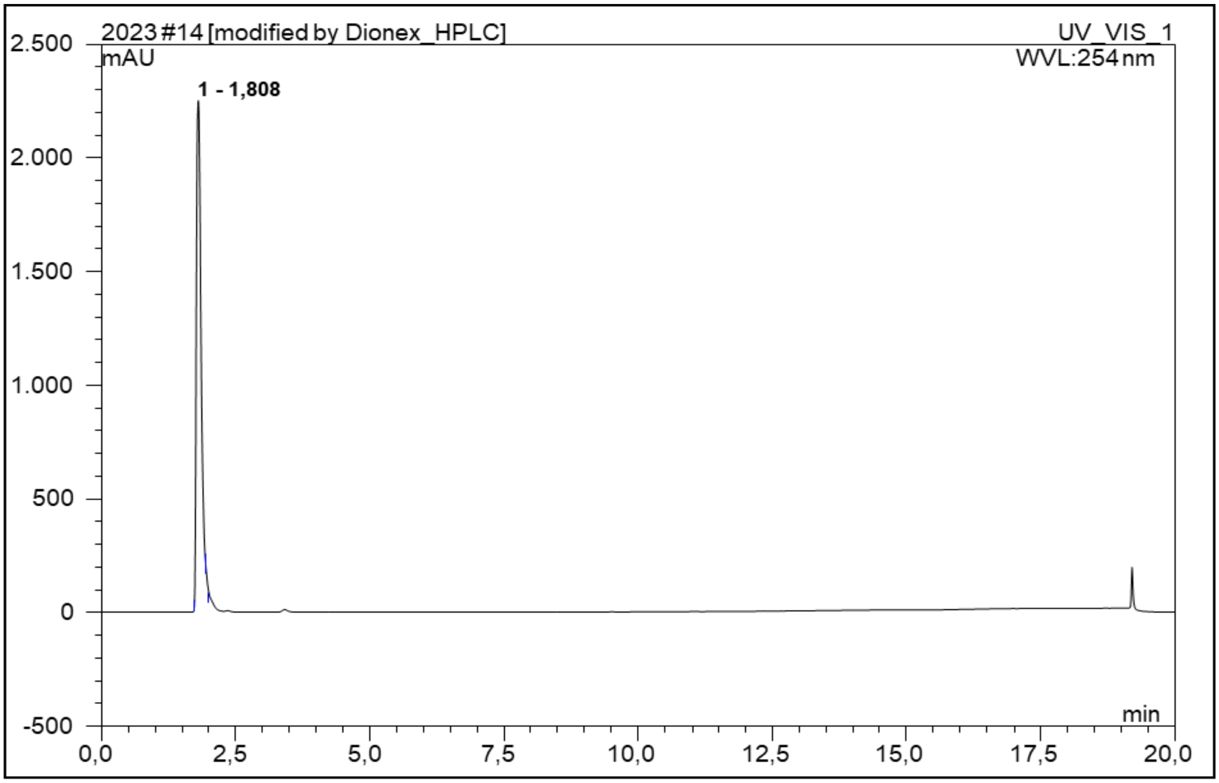

Figure S6. Analytical HPLC spectrum of **SS-SAH.**

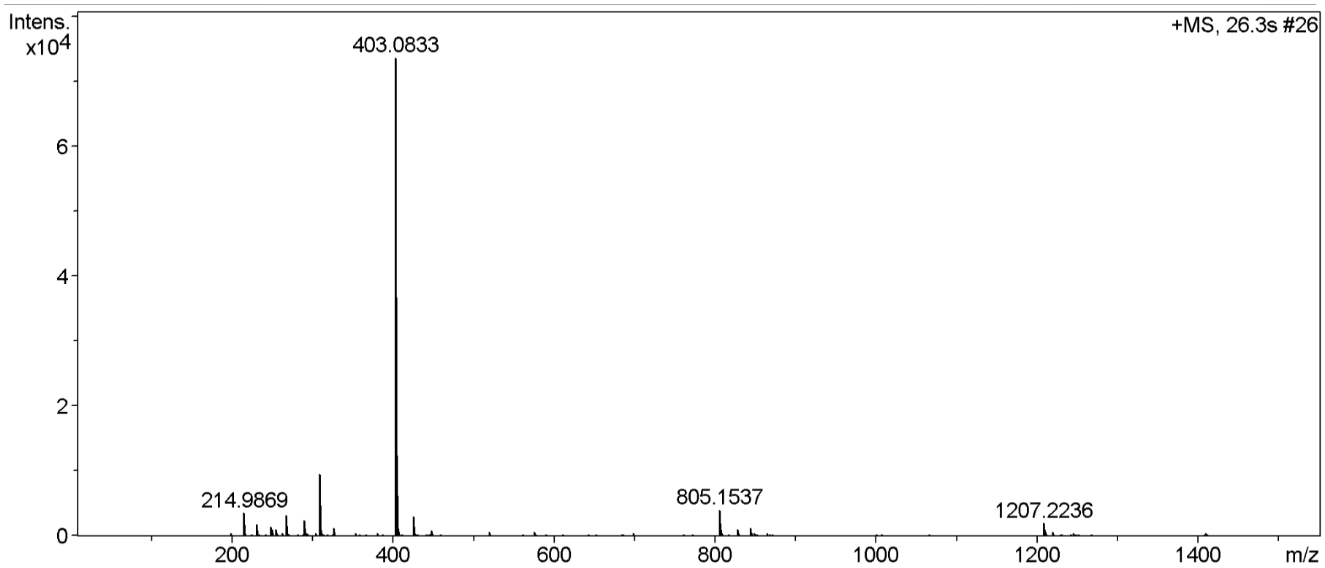

Figure S7. ESI-HR-MS spectrum of **SS-SAH.**

Degradation of SS-SAH in the presence of DTT and GSH

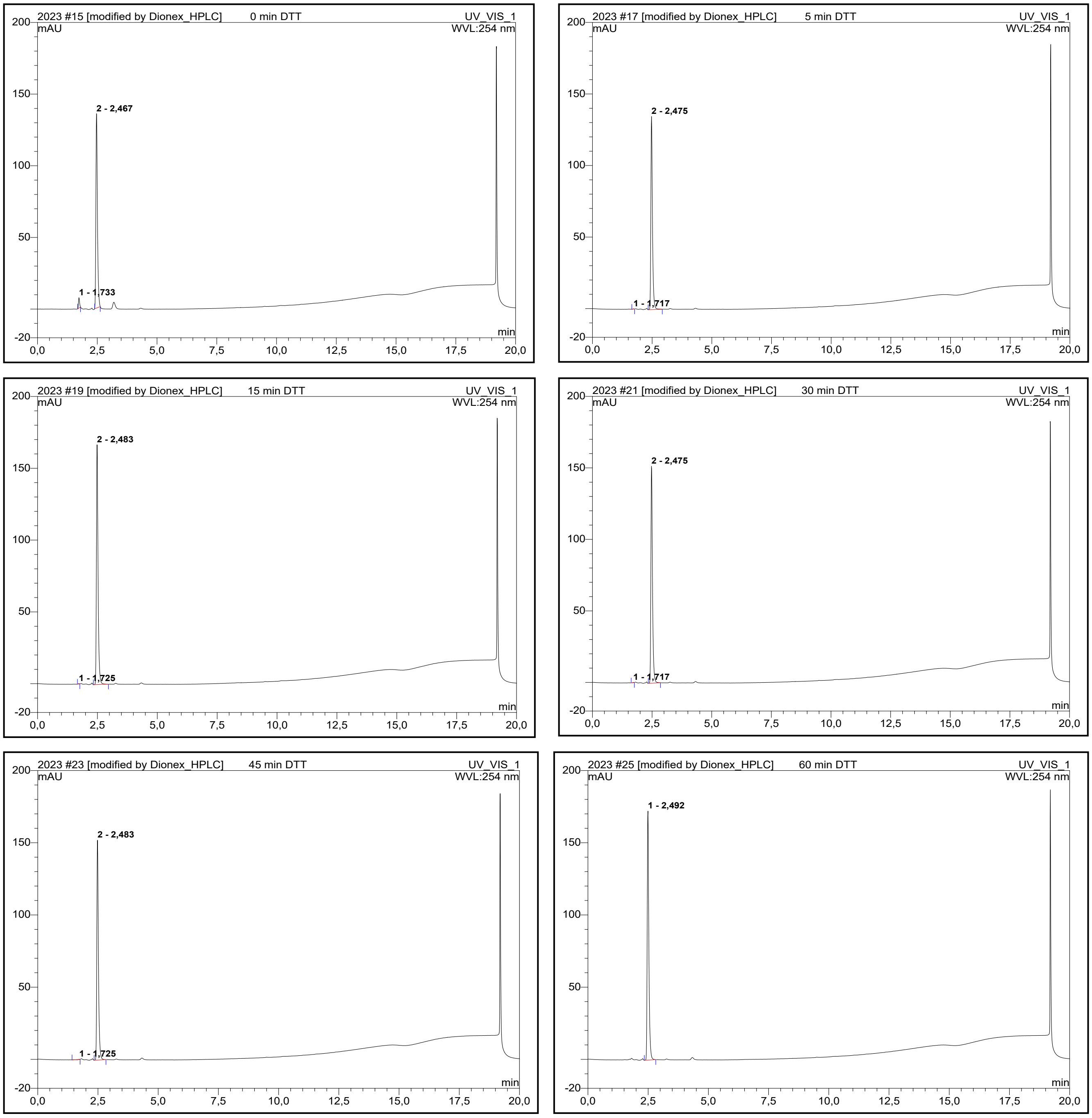

Figure S8. Degradation of 100 µM of **SS-SAH** by 1 mM of DTT within 60 minutes.

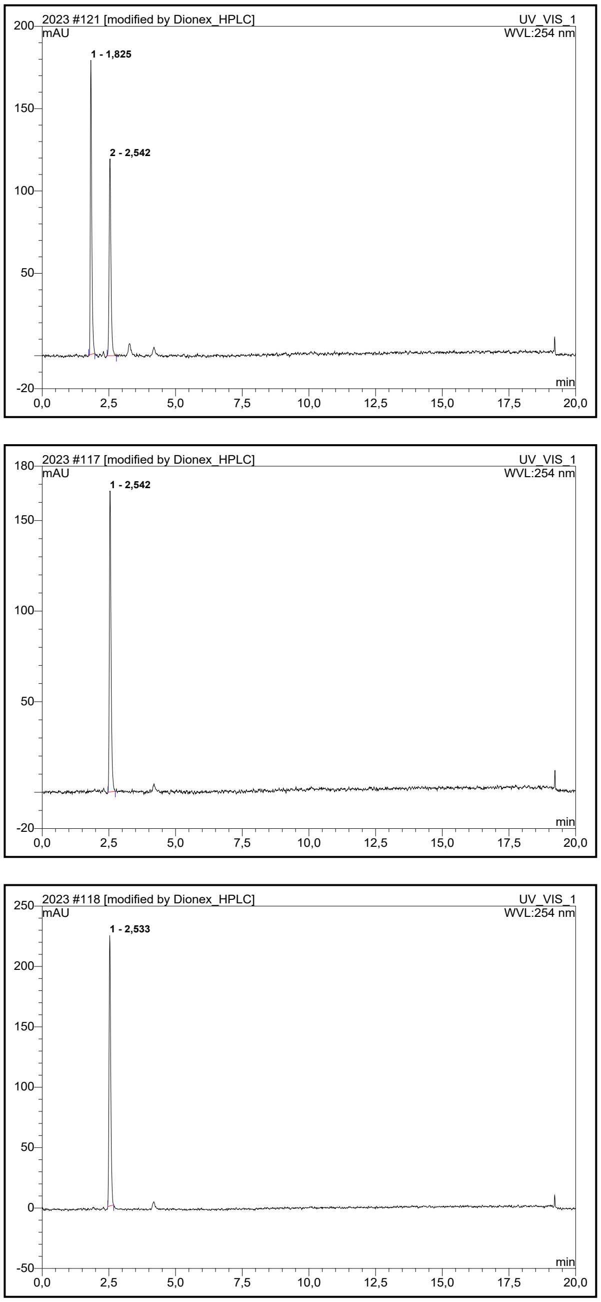

Figure S9. Degradation of 100μM of **SS-SAH** by 500μM of DTT within 60 minutes. Top) 0 min, Middle) 15 min, Bottom) 60 min.

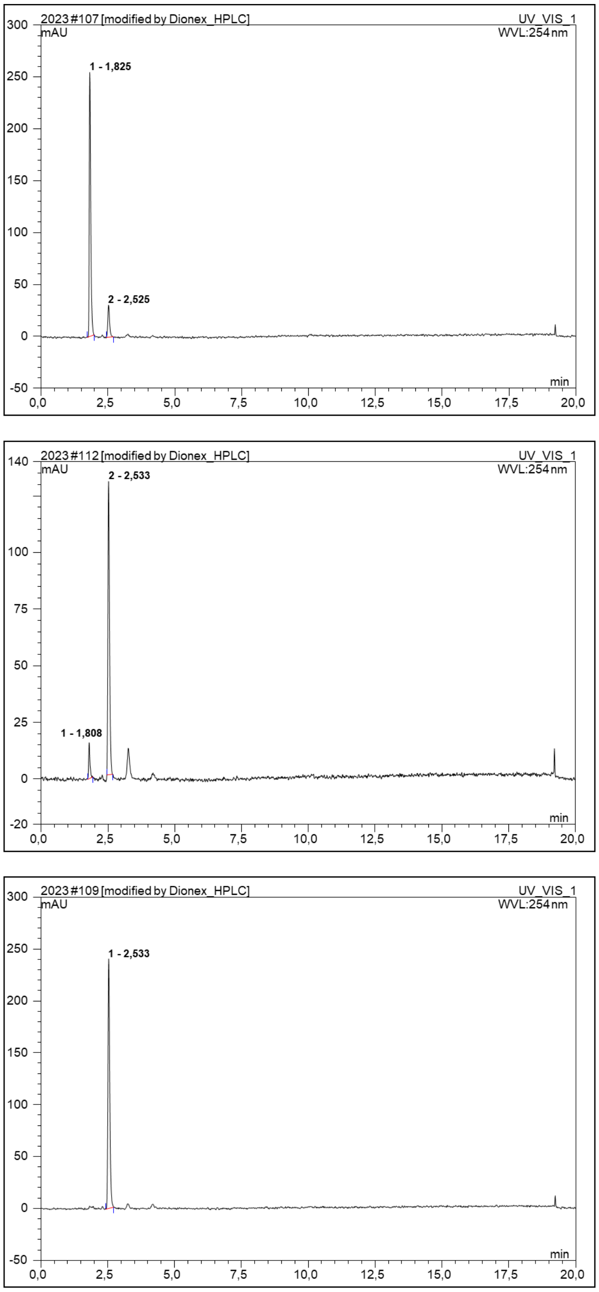

Figure S10. Degradation of 100 µM of **SS-SAH** by 200 µM of DTT within 60 minutes. Top) 0 min, Middle) 15 min, Bottom) 60 min.

a)

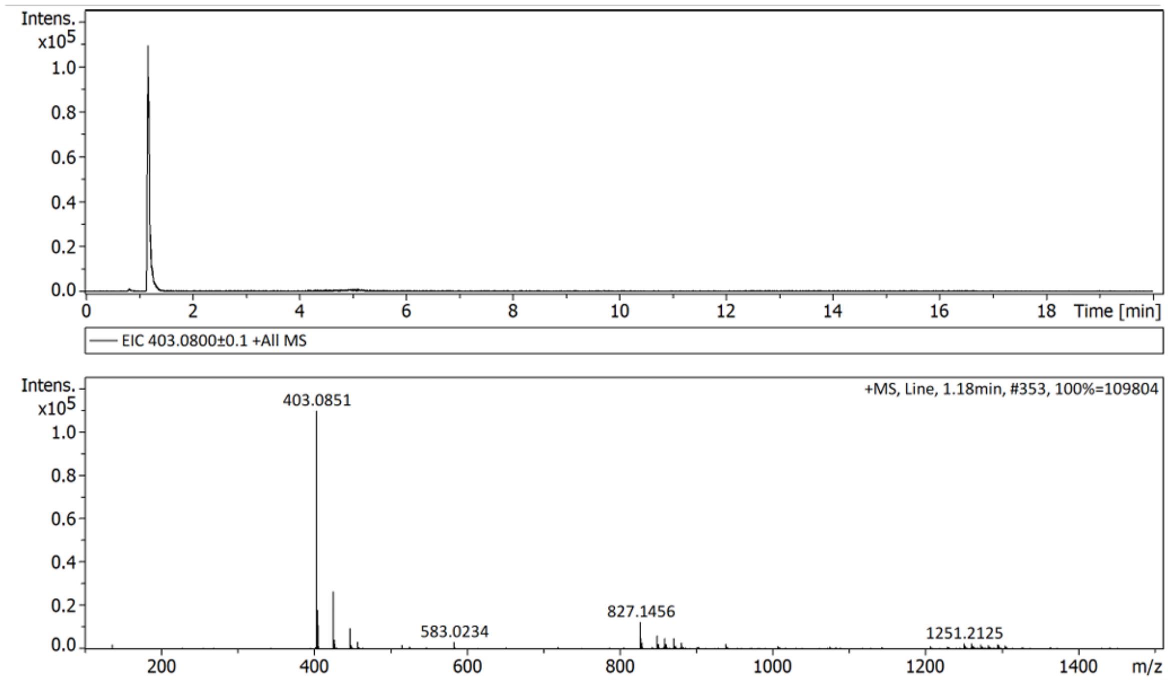

b)

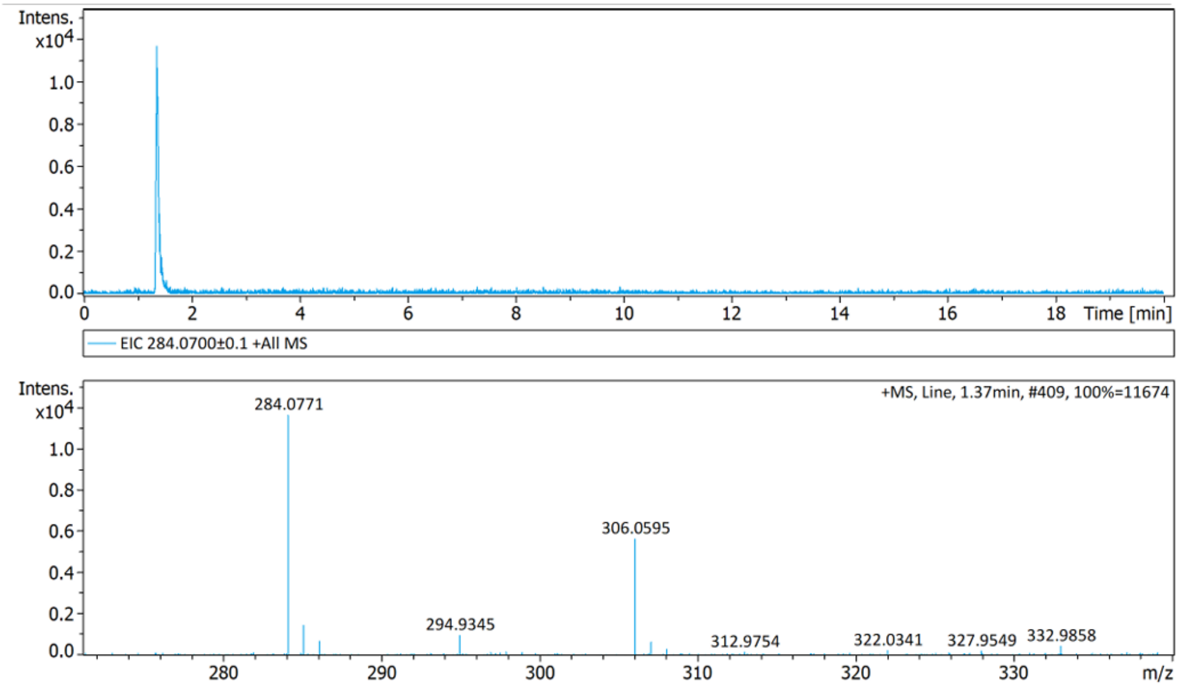

Figure S11. LC-MS data showing a) 100 µM of **SS-SAH** and b) 100 µM of **SS-SAH** in the presence of 1 mM DTT after 1 hour.

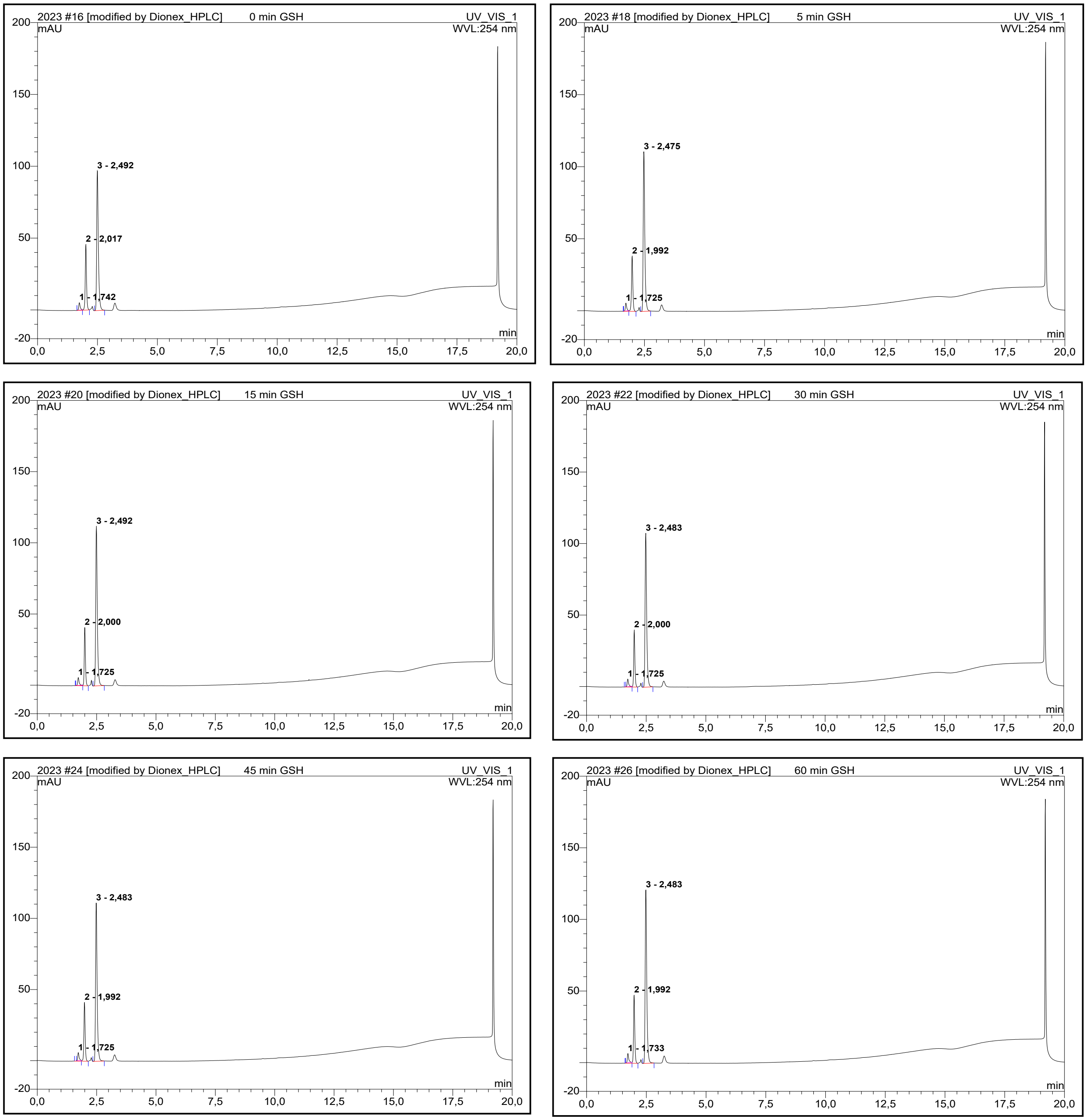

Figure S12. Degradation of 100 µM of **SS-SAH** with 1 mM of GSH within 60 minutes.

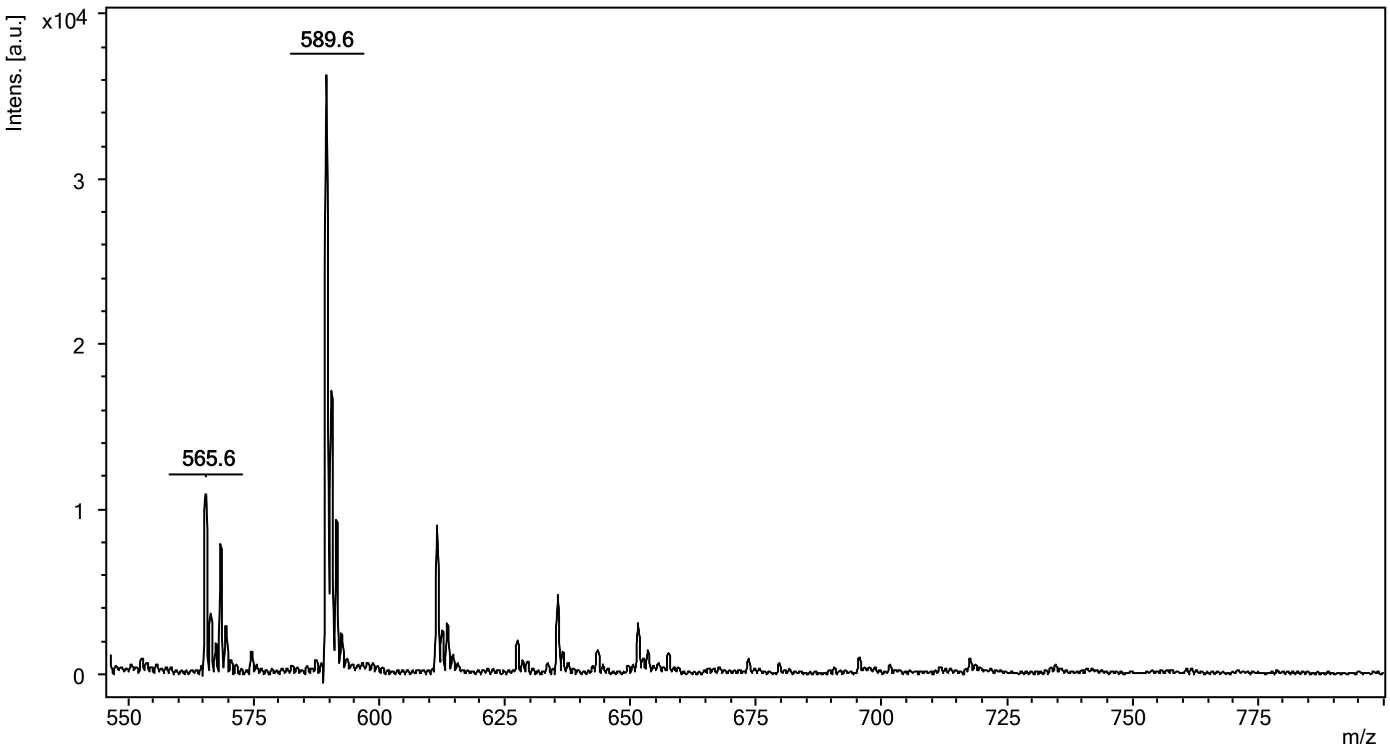

Figure S13. MALDI-TOF spectrum indicating the presence of a disulfide comprised of thiol **3** and GSH, here seen as $[M+H]^+$, 589.6 m/z. 565.6 m/z corresponds to the matrix.

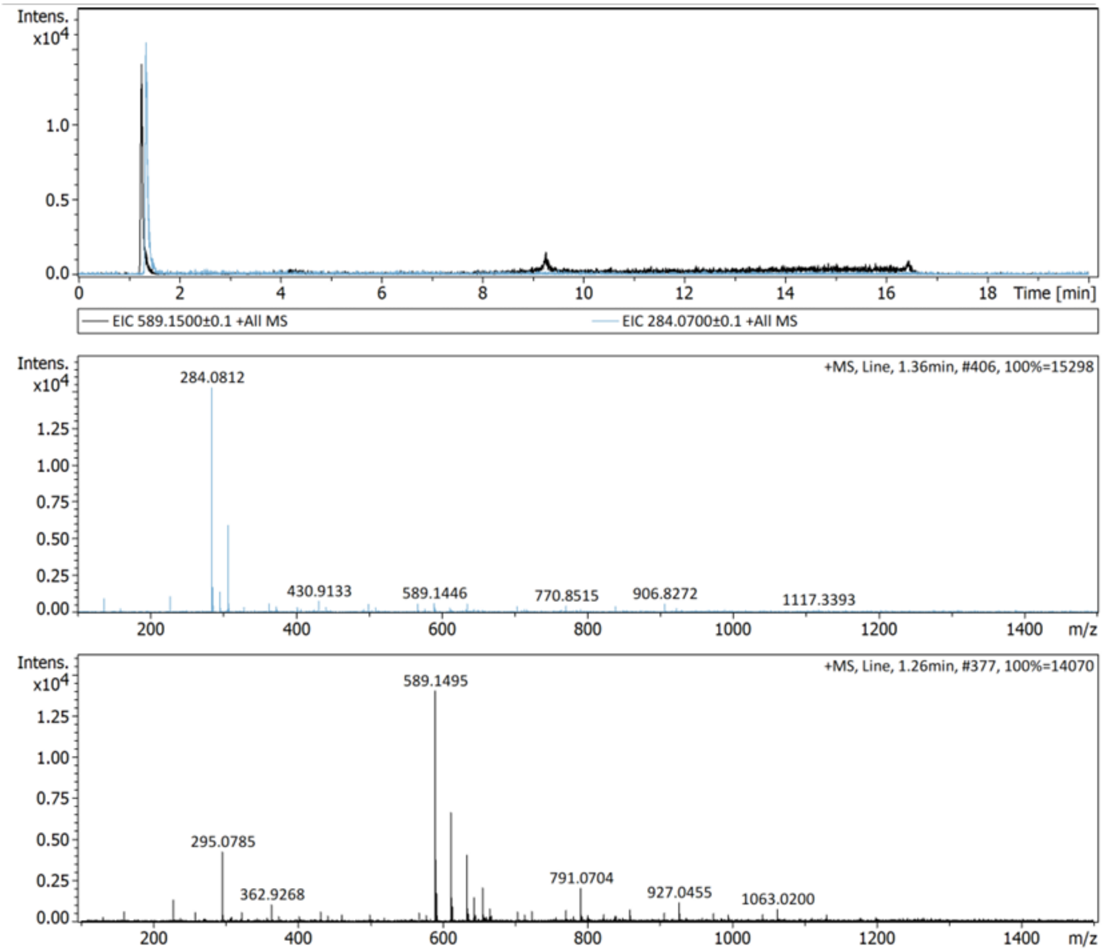

Figure S14. LC-MS data showing the reduction of 100 µM of **SS-SAH** by 1 mM glutathione (GSH) after 1 hour. Top) the chromatogram of the formed thiol **3** and **3**-GSH adduct. Middle) MS data of thiol **3**. Bottom) MS data of the **3**-GSH adduct (the structure is shown in Figure S15).

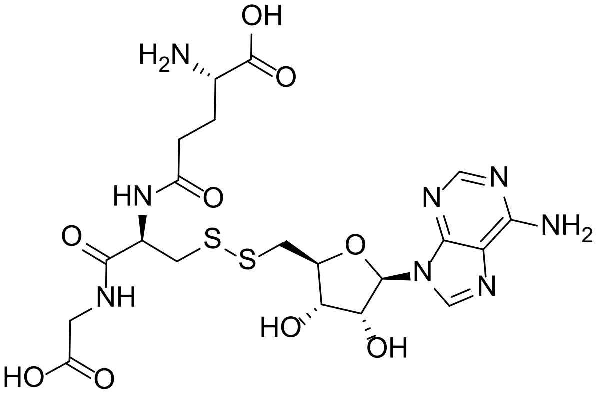

Figure S15. Structure of an asymmetric disulfide comprised of GSH and thiol **3.**

Inhibition of SETD8 by SAH

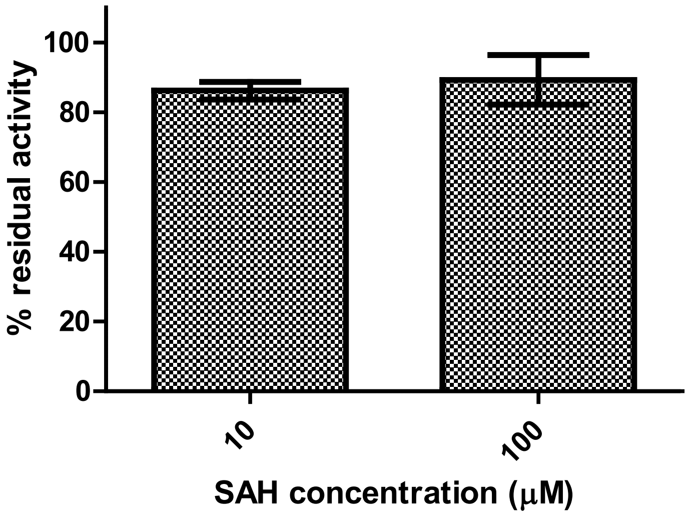

Figure S16. Single point inhibition assay of SAH against SETD8 in the presence of 10 and 100 μM of SAH**.** Estimated IC_{50} > 100 μM. Assay was performed in independent replicates (n = 2) and all points include SEM.

Docking and molecular dynamics

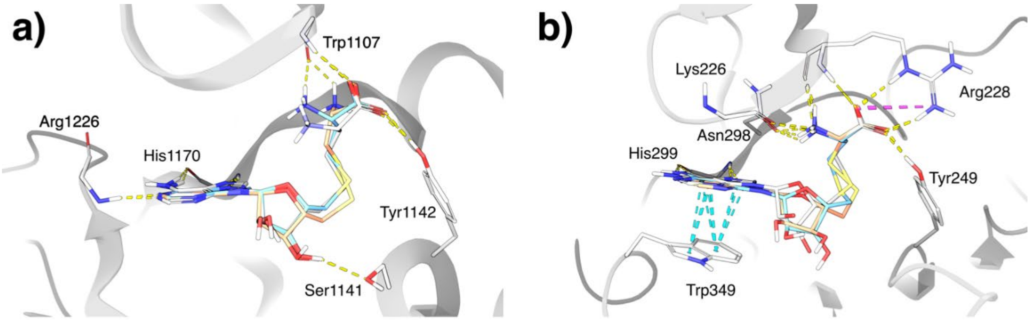

Figure S17. Restrained docking of SAH and **SS-SAH** to the binding site of a) GLP (PDB ID: 3HNA) and, b) SETD8 (1ZKK). Orange: docked **SS-SAH**, blue: docked SAH and grey: crystal structure. Images were made in Maestro from the Schrödinger Suite 2019-1 (https://www.schrodinger.com/platform/products/maestro/).

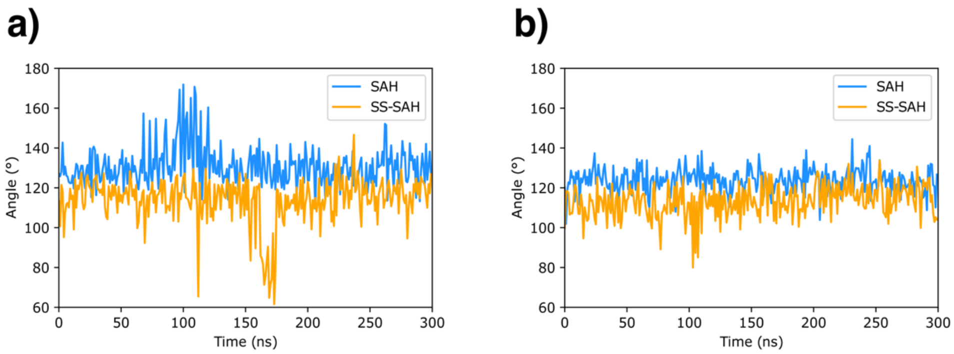

Figure S18. Angle of the dihedral defined by the S-S bond in **SS-SAH** (the C-H bond in SAH) during the MD simulations to a) GLP and b) SETD8.

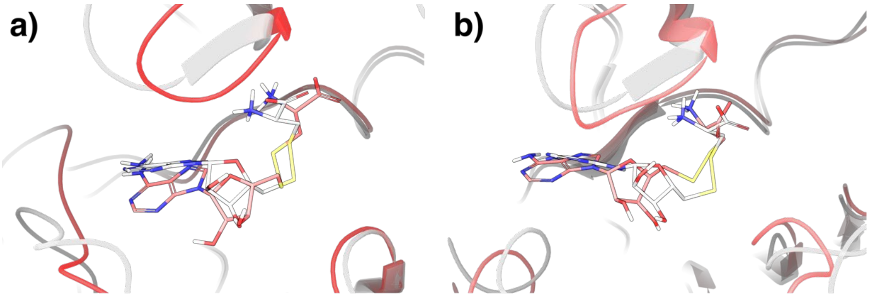

Figure S19. a) Snapshot at 165 ns from the MD simulation of **SS-SAH** bound to GLP (red) compared to the final snapshot at 300 ns. b) Snapshot at 105 ns from the MD simulation of **SS-SAH** bound to SETD8 (red) compared to the final snapshot at 300 ns. Images were made in Maestro from the Schrödinger Suite 2019-1 (https://www.schrodinger.com/platform/products/maestro/).

Percentages of histone H3K9 methylation states during deactivation of SS-SAH with DTT

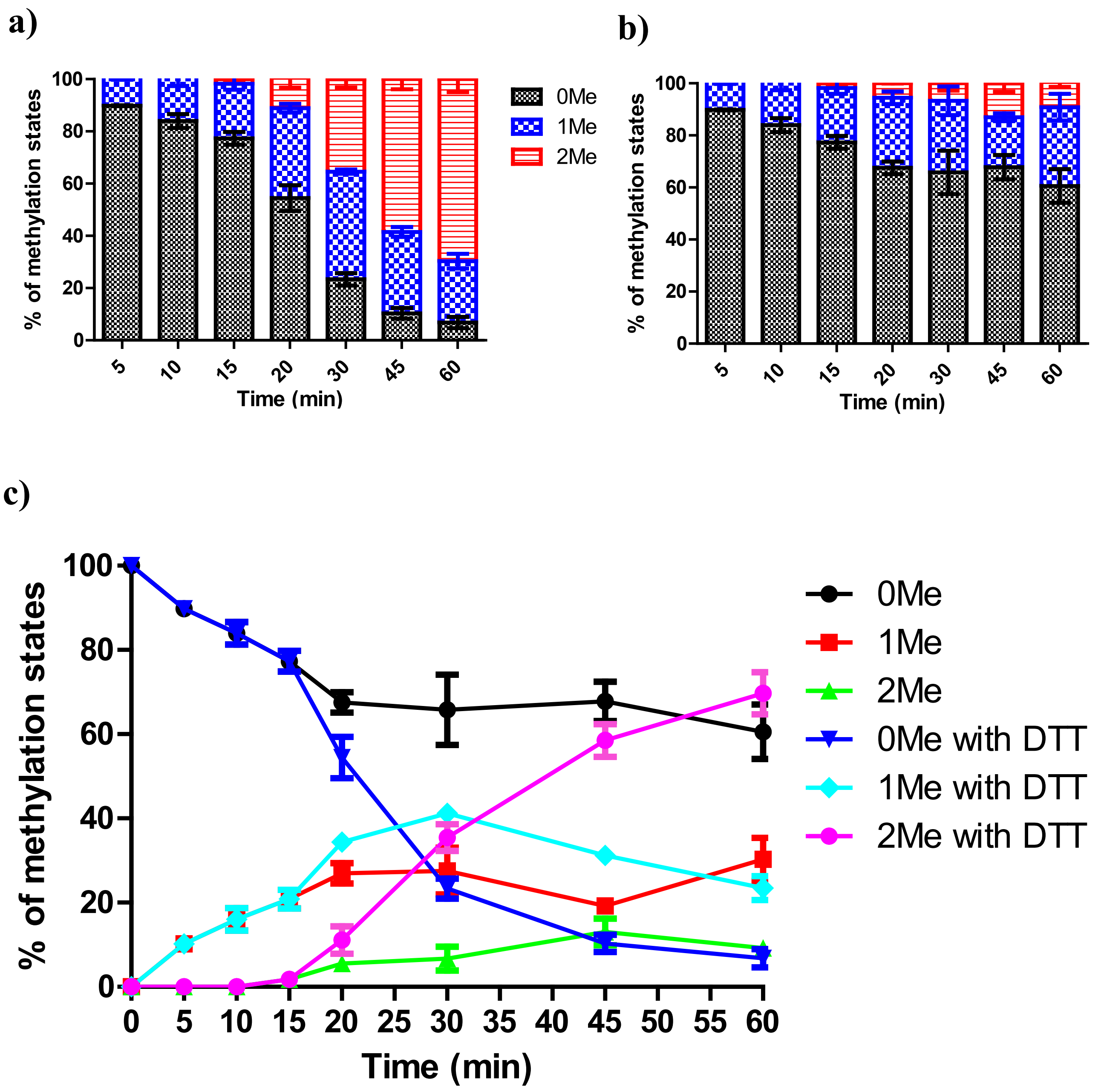

Figure S20. Percentage of methylation states of histone H3K9 over time catalyzed by GLP in the presence of 100 µM of **SS-SAH.** a) With addition of 500 µM of DTT at 15 min. b) With no addition of DTT. c) Comparison of methylation state percentage over time in presence and absence of DTT. Assay conditions: 200 nM GLP, 5 µM histone H3K9 peptide, 20 µM SAM. Assays were performed in independent replicates (n = 2) and all points include SEM.

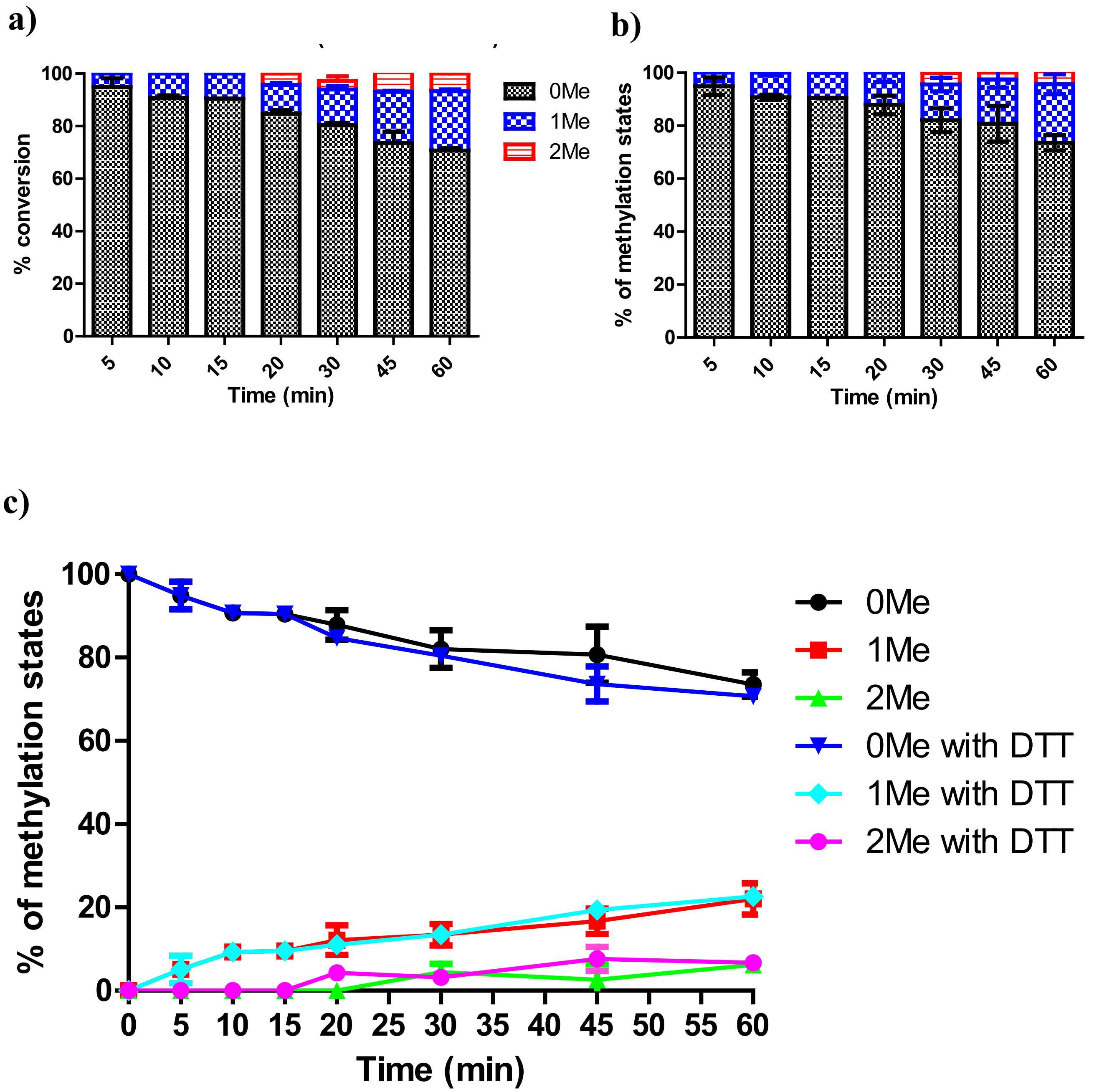

Figure S21. Percentage of methylation states of histone H3K9 over time catalyzed by GLP in the presence of 100 μM of SAH**.** a) With addition of 500 μM of DTT at 15 min. b) With no addition of DTT. c) Comparison of methylation state percentage over time in presence and absence of DTT. Assay conditions: 200 nM GLP, 5 μM histone H3K9 peptide, 20 μM SAM. Assays were performed in independent replicates (n = 2) and all points include SEM.

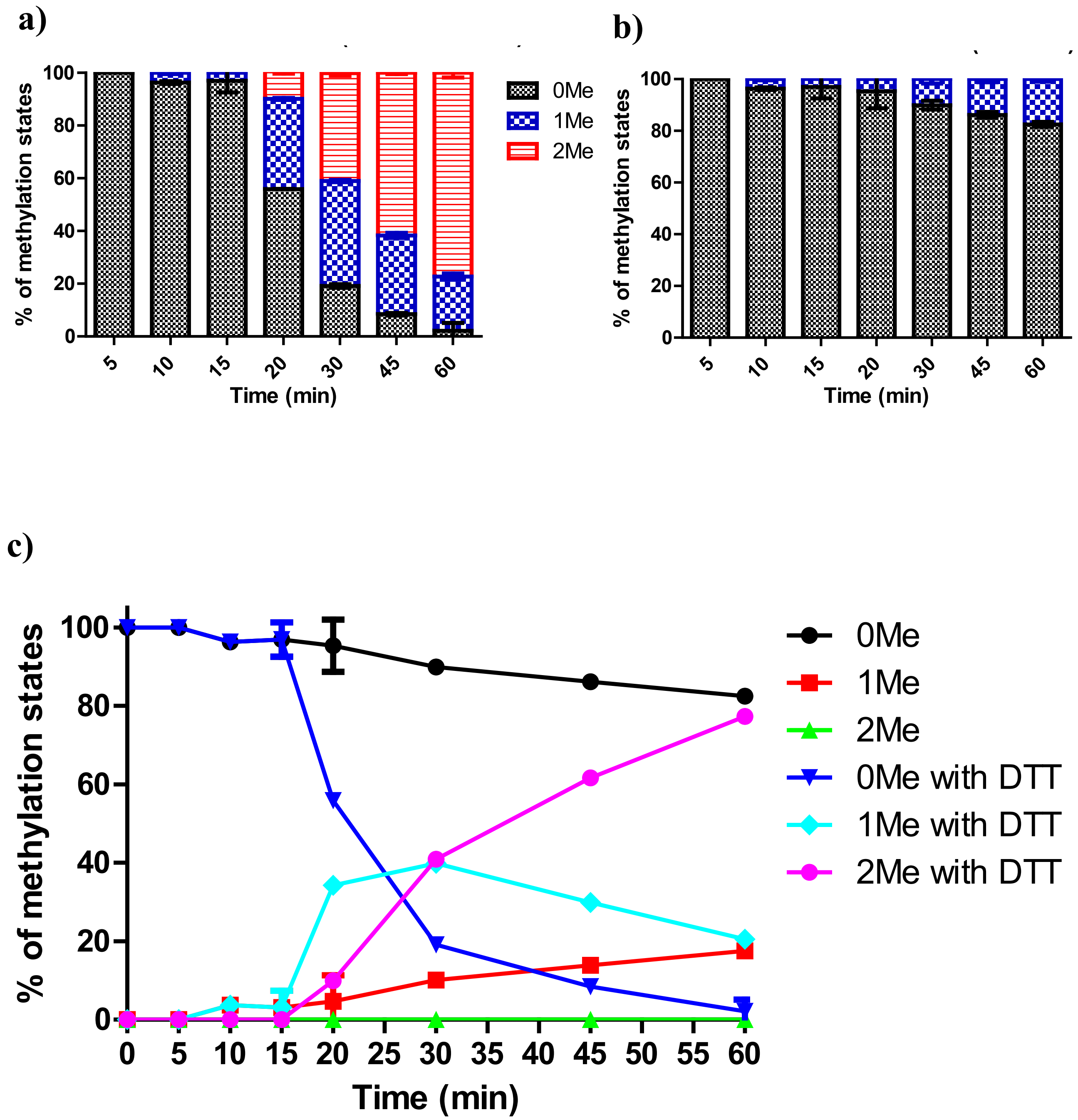

Figure S22. Percentage of methylation states of histone H3K9 over time catalyzed by GLP in the presence of 500 μM of **SS-SAH.** a) With addition of 2.5 mM of DTT at 15 min. b) With no addition of DTT. c) Comparison of methylation state percentage over time in presence and absence of DTT. Assay conditions: 200 nM GLP, 5 μM histone H3K9 peptide, 20 μM SAM. Assays were performed in independent replicates (n = 2) and all points include SEM.

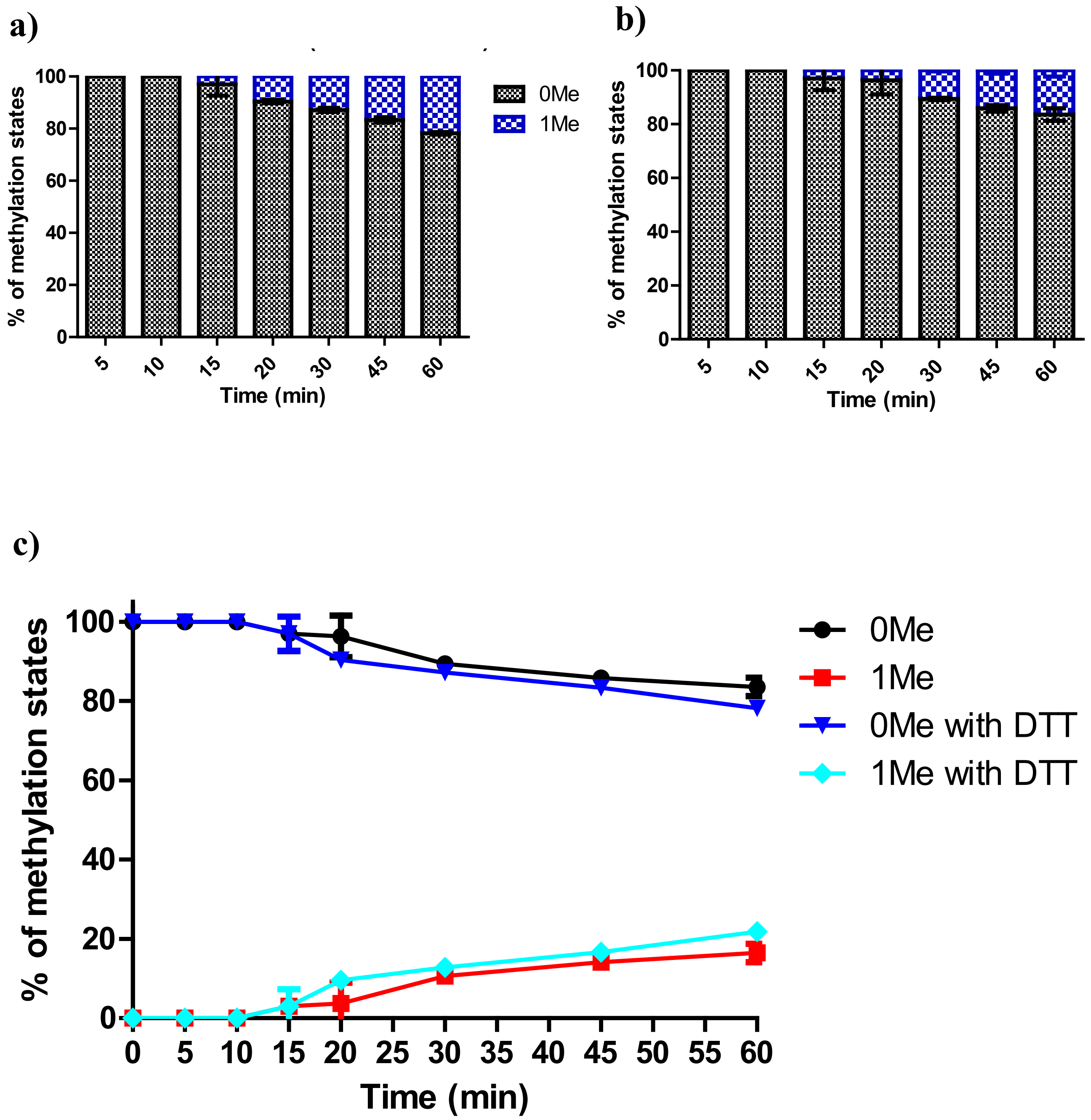

Figure S23. Percentage of methylation states of histone H3K9 over time catalyzed by GLP in the presence of 500 µM of SAH**.** a) With addition of 2.5 mM of DTT at 15 min. b) With no addition of DTT. c) Comparison of methylation state percentage over time in presence and absence of DTT. Assay conditions: 200 nM GLP, 5 µM histone H3K9 peptide, 20 µM SAM. Assays were performed in independent replicates (n = 2) and all points include SEM.

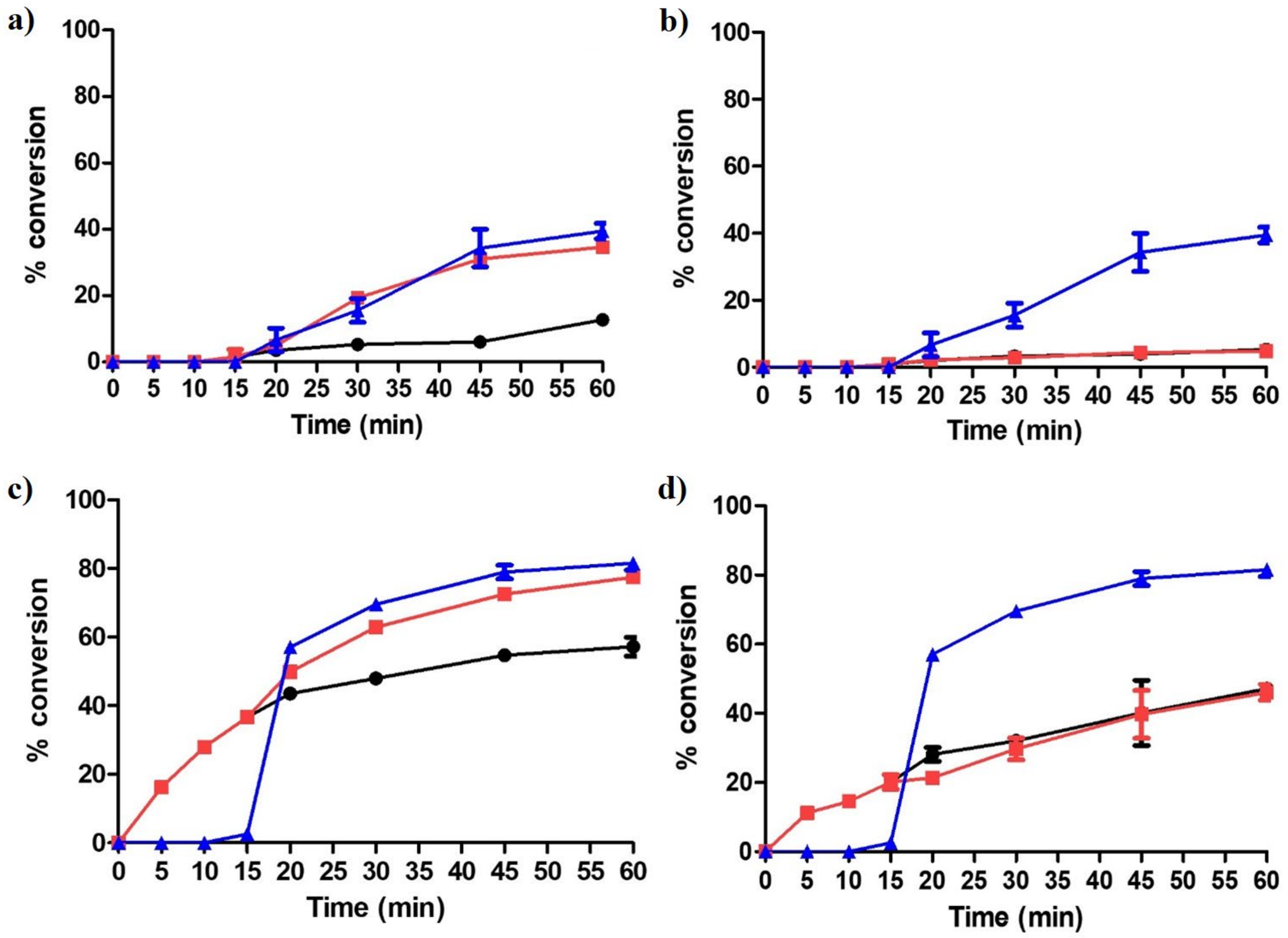

Figure S24. Methylation of histone H3K9 (5 µM) by GLP over time in the presence of 100 µM of **SS-SAH** or 100 µM of SAH and addition of 500 µM of DTT. a) **SS-SAH** and 100 nM of GLP. b) SAH and 100 nM of GLP. c) **SS-SAH** and 500 nM of GLP. d) SAH and 500 nM of GLP. – Black: Conversion in presence of inhibitor, Red: Conversion in presence of inhibitor and addition of DTT at 15 min., Blue: Conversion without inhibitor, initiated with addition of SAM (20 µM) at 15min. Assays were performed in independent replicates (n = 2) and all points include SEM.

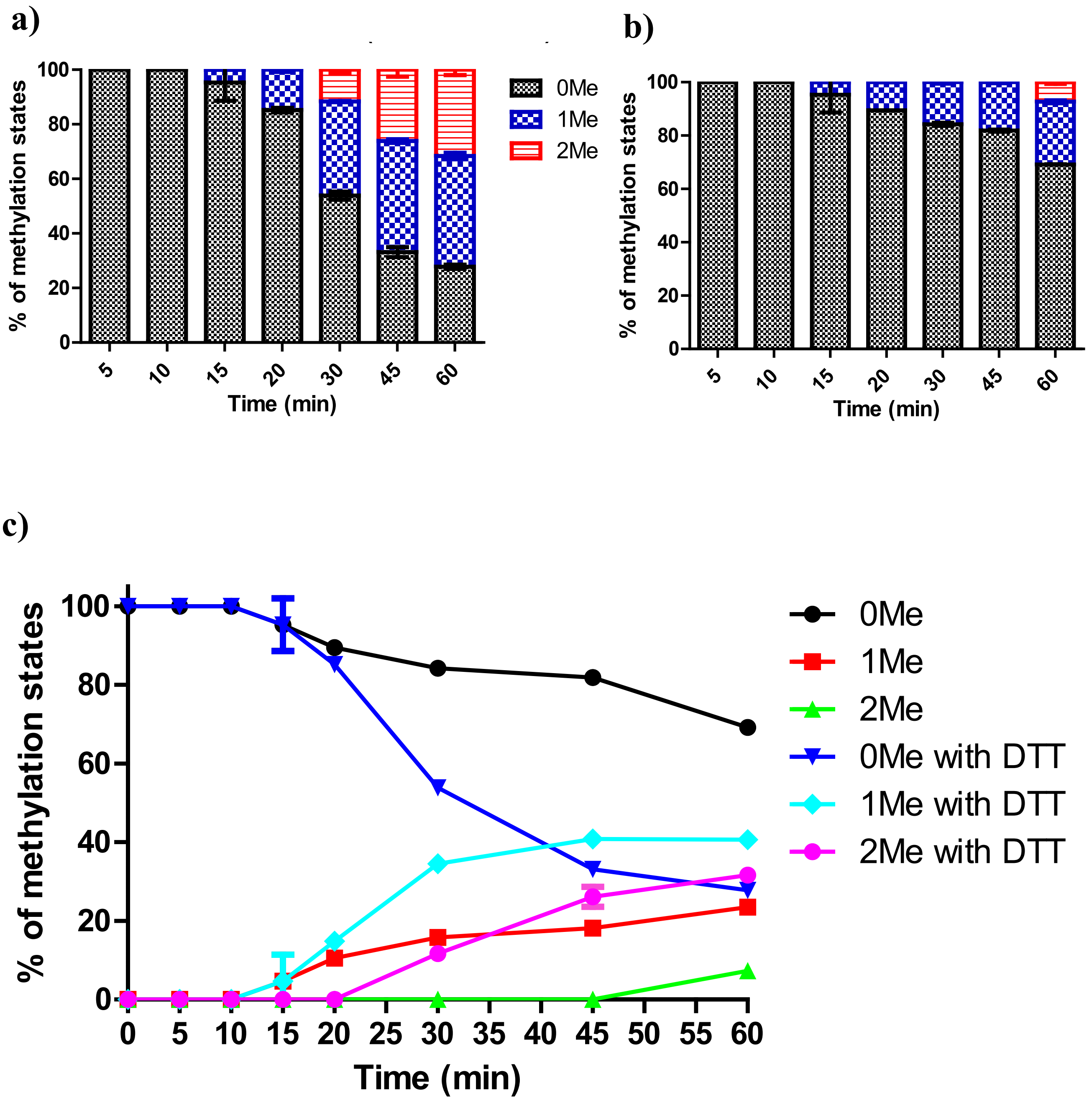

Figure S25. Percentage of methylation states of histone H3K9 over time catalyzed by GLP in the presence of 100 µM of **SS-SAH.** a) With addition of 500 µM of DTT at 15 min. b) With no addition of DTT. c) Comparison of methylation state percentage over time in presence and absence of DTT. Assay conditions: 100 nM GLP, 5 µM histone H3K9 peptide, 20 µM SAM. Assays were performed in independent replicates (n = 2) and all points include SEM.

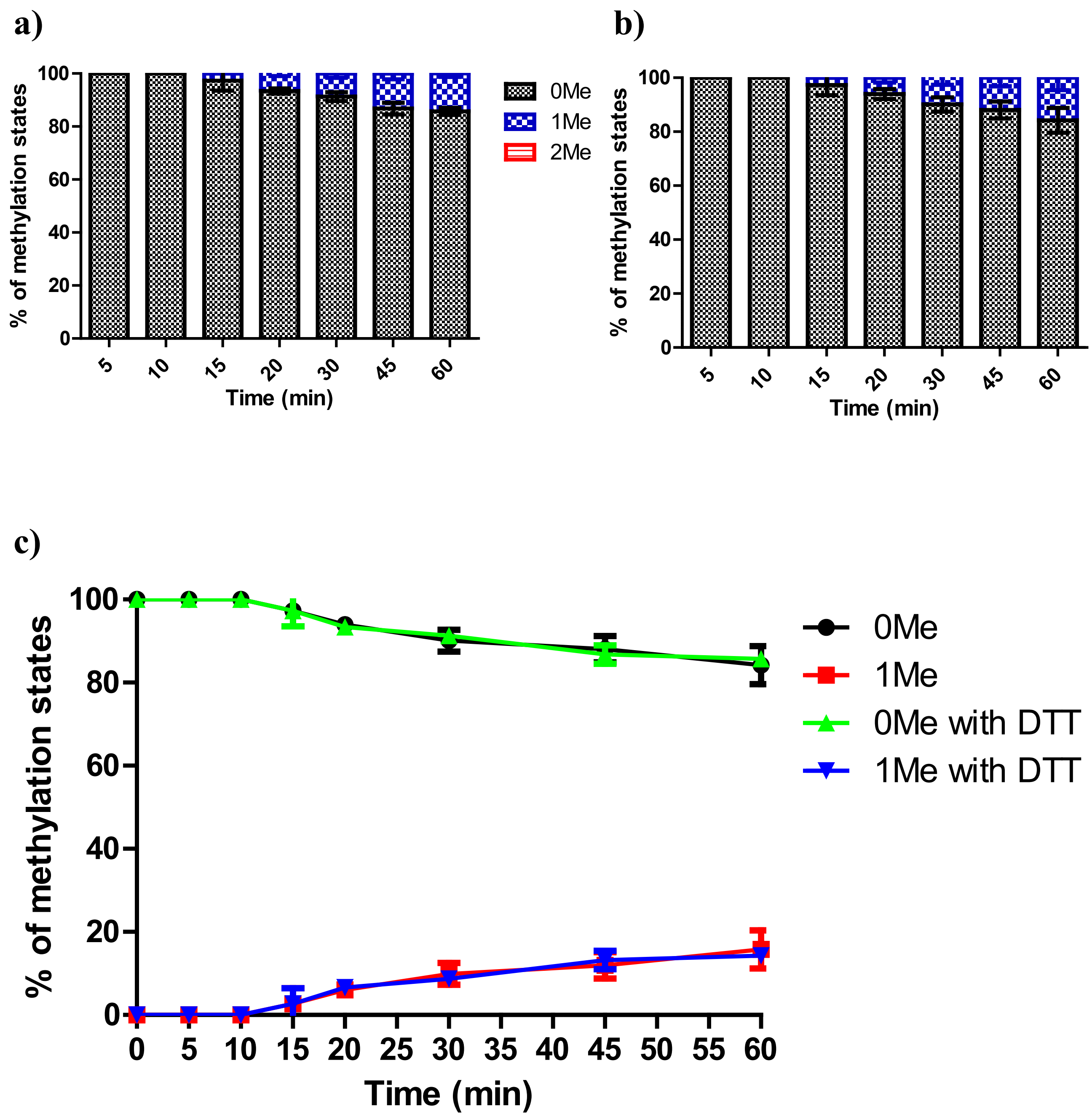

Figure S26. Percentage of methylation states of histone H3K9 over time catalyzed by GLP in the presence of 100 µM of SAH**.** a) With addition of 500 µM of DTT at 15 min. b) With no addition of DTT. c) Comparison of methylation state percentage over time in presence and absence of DTT. Assay conditions: 100 nM GLP, 5 µM histone H3K9 peptide, 20 µM SAM. Assays were performed in independent replicates (n = 2) and all points include SEM.

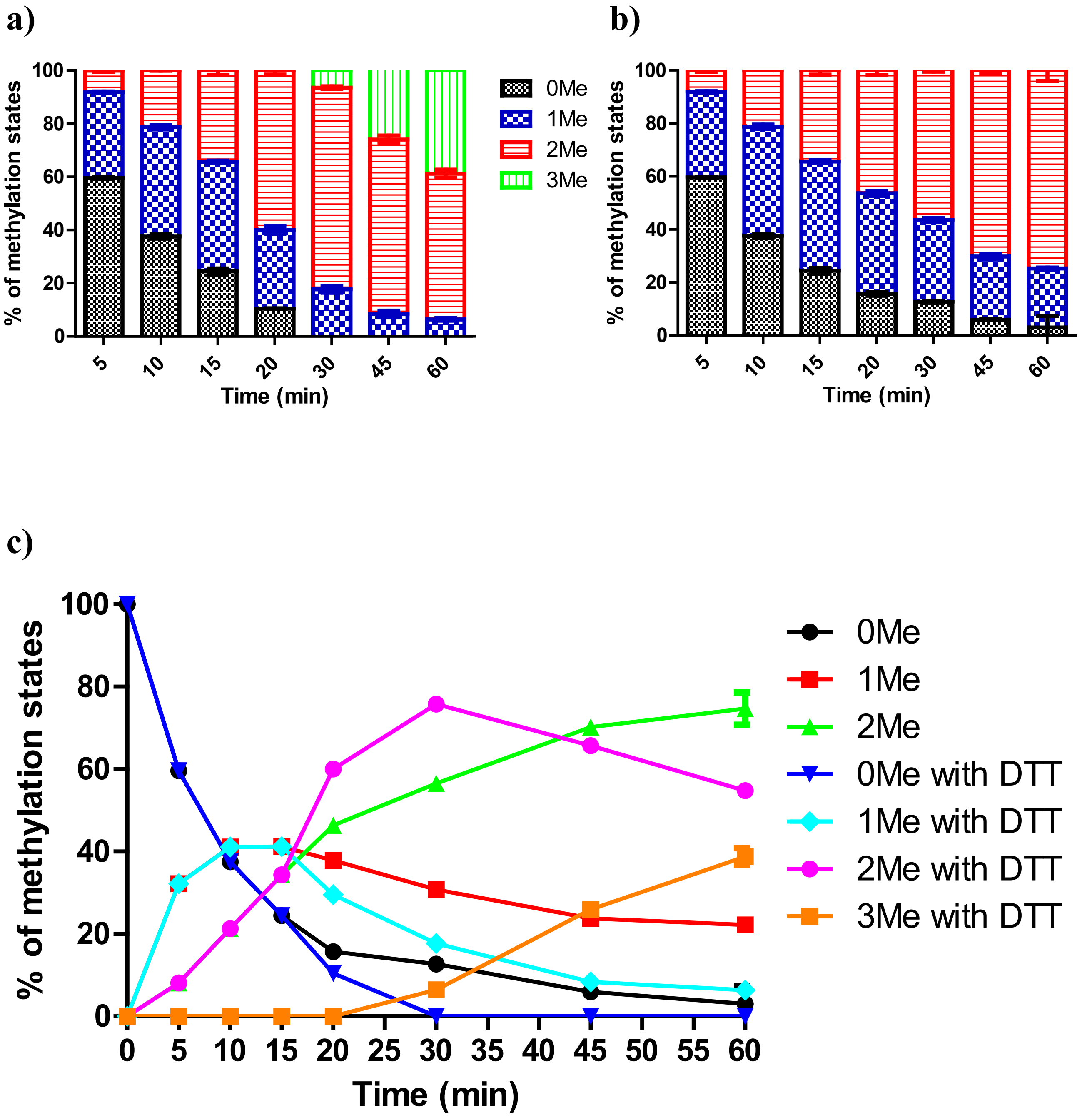

Figure S27. Percentage of methylation states of histone H3K9 over time catalyzed by GLP in the presence of 100 µM of **SS-SAH.** a) With addition of 500 µM of DTT at 15 min. b) With no addition of DTT. c) Comparison of methylation state percentage over time in presence and absence of DTT. Assay conditions: 500 nM GLP, 5 µM histone H3K9 peptide, 20 µM SAM. Assays were performed in independent replicates (n = 2) and all points include SEM.

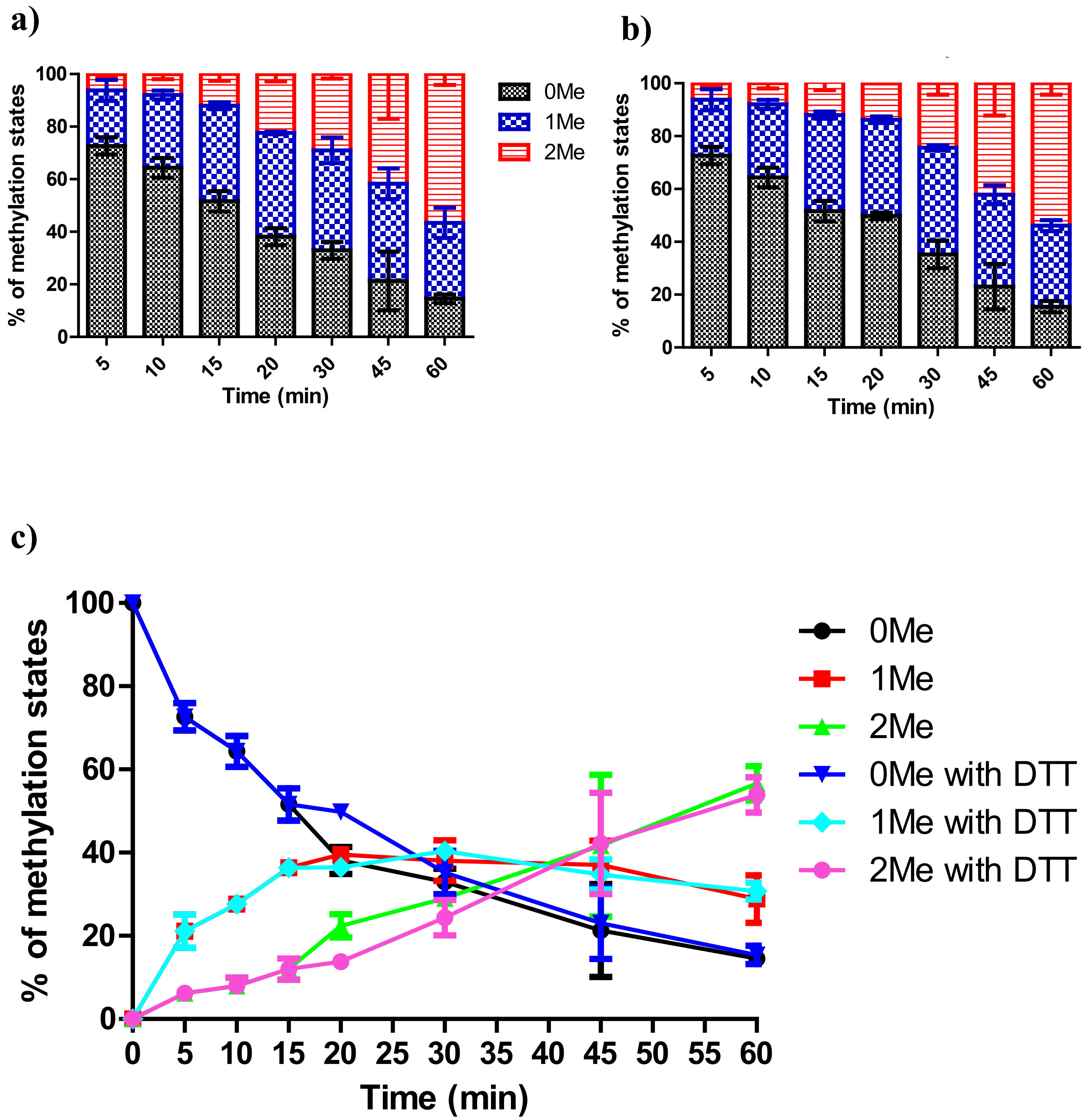

Figure S28. Percentage of methylation states of histone H3K9 over time catalyzed by GLP in the presence of 100 µM of SAH**.** a) With addition of 500 µM of DTT at 15 min. b) With no addition of DTT. c) Comparison of methylation state percentage over time in presence and absence of DTT. Assay conditions: 500 nM GLP, 5 µM histone H3K9 peptide, 20 µM SAM. Assays were performed in independent replicates (n = 2) and all points include SEM.

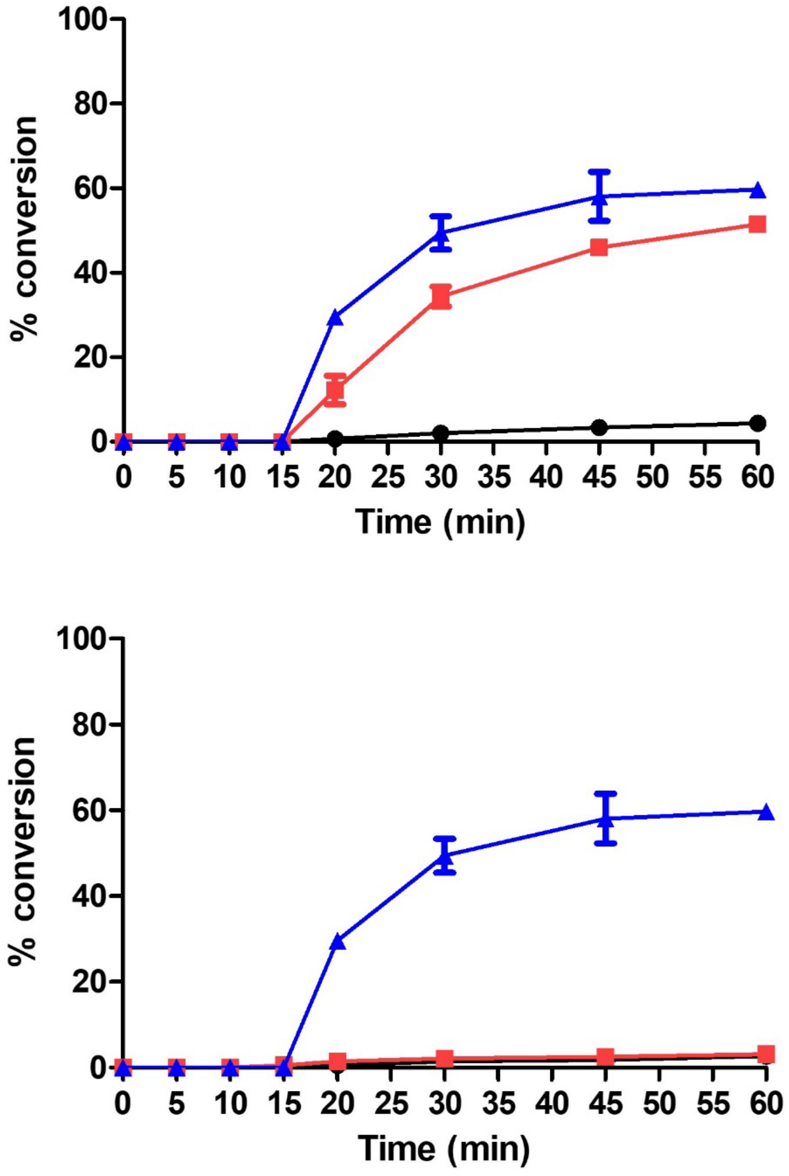

Figure S29. Methylation of histone H3K9 by GLP over time in the presence of **SS-SAH** (top) or SAH (bottom). Black: Conversion in presence of inhibitor, Red: Conversion in presence of inhibitor and addition of 1 mM (above) and 2.5 mM (bottom) of DTT at 15 min., Blue: Conversion without inhibitor, initiated with addition of SAM at 15 min. Assay conditions: 200 nM GLP, 5 µM histone H3K9 peptide, 20 µM SAM. Assays were performed in independent replicates (n = 2) and all points include SEM.

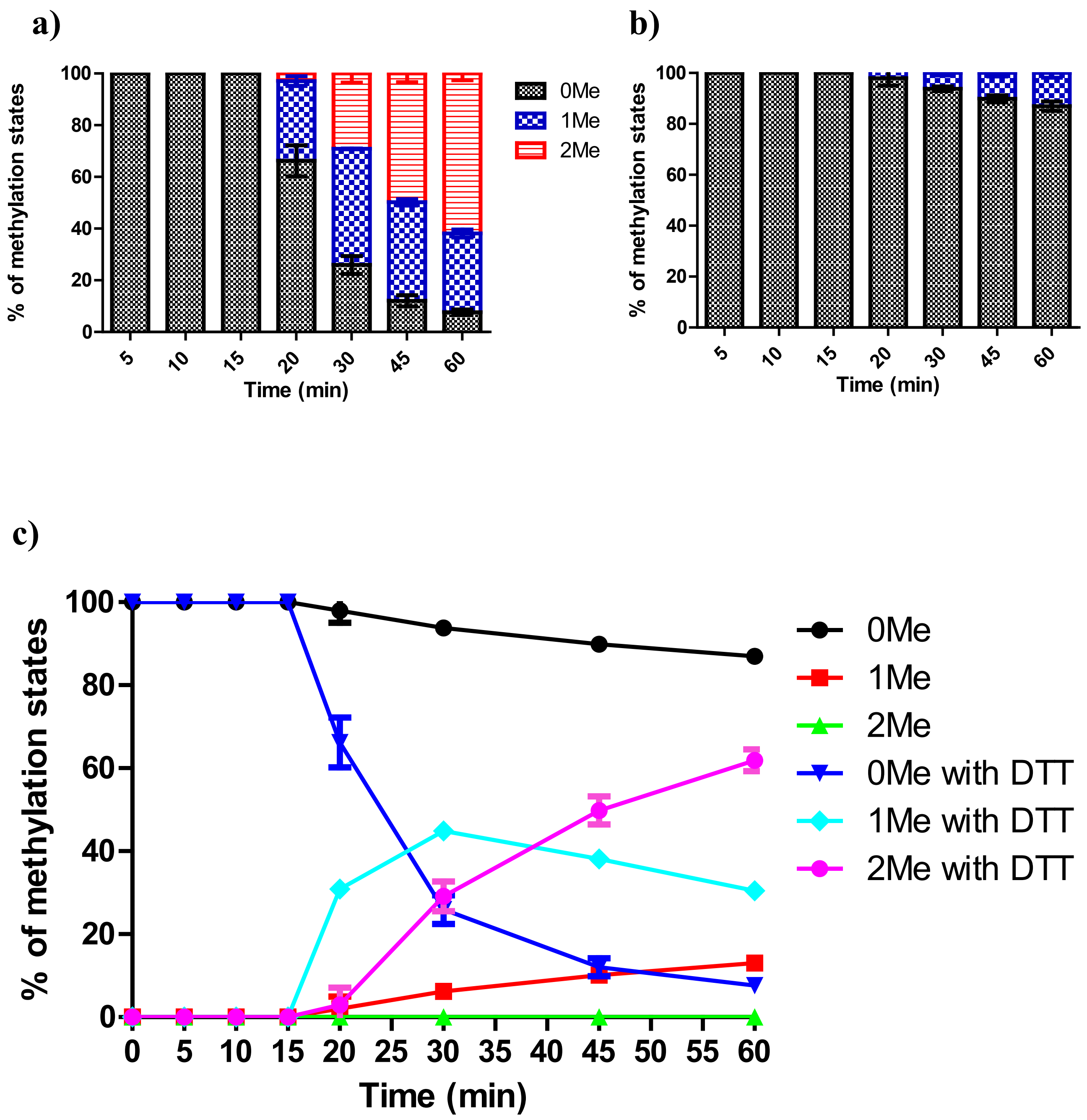

Figure S30. Percentage of methylation states of histone H3K9 over time catalyzed by GLP in the presence of 500 µM of **SS-SAH.** a) With addition of 1 mM of DTT at 15 min. b) With no addition of DTT. c) Comparison of methylation state percentage over time in presence and absence of DTT. Assay conditions: 200 nM GLP, 5 µM histone H3K9 peptide, 20 µM SAM. Assays were performed in independent replicates (n = 2) and all points include SEM.

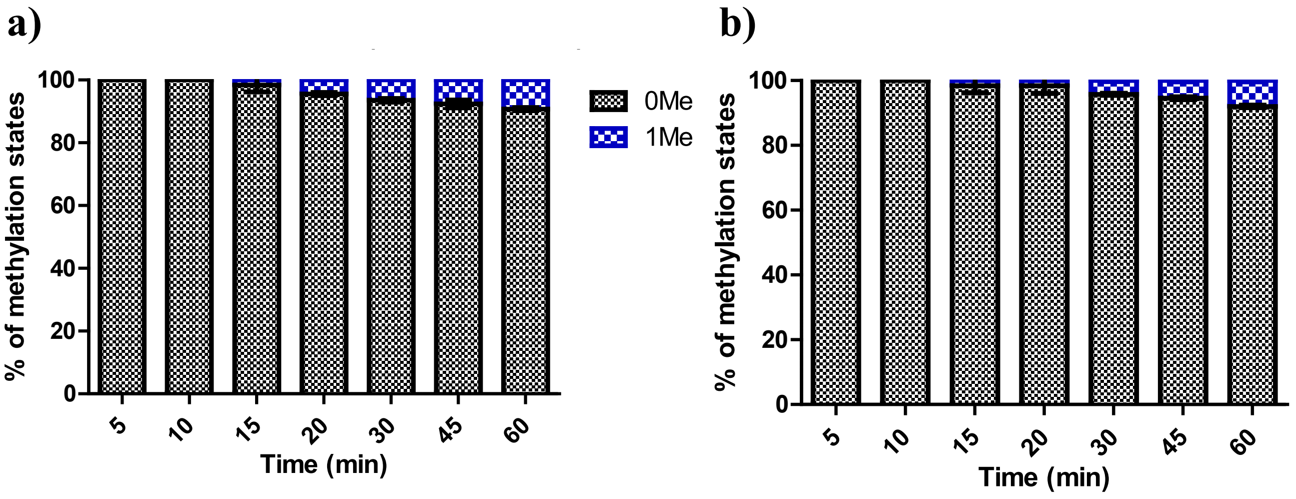

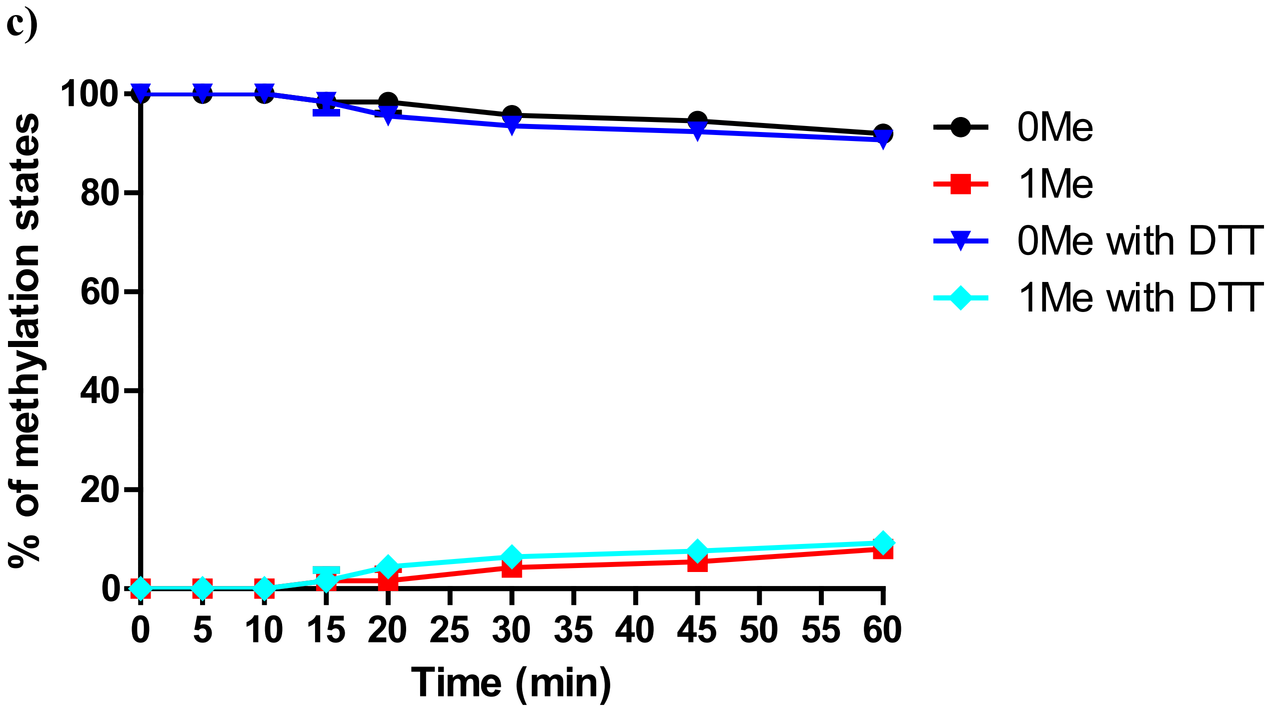

Figure S31. Percentage of methylation states of histone H3K9 over time catalyzed by GLP in the presence of 500 µM of SAH**.** a) With addition of 1 mM of DTT at 15 min. b) With no addition of DTT. c) Comparison of methylation state percentage over time in presence and absence of DTT. Assay conditions: 200 nM GLP, 5 µM histone H3K9 peptide, 20 µM SAM. Assays were performed in independent replicates (n = 2) and all points include SEM.
